# *Brucella abortus* Induces the Premature Death of Human Neutrophils through the Action of Its Lipopolysaccharide

**DOI:** 10.1371/journal.ppat.1004853

**Published:** 2015-05-06

**Authors:** Elías Barquero-Calvo, Ricardo Mora-Cartín, Vilma Arce-Gorvel, Juana L. de Diego, Carlos Chacón-Díaz, Esteban Chaves-Olarte, Caterina Guzmán-Verri, Andre G. Buret, Jean-Pierre Gorvel, Edgardo Moreno

**Affiliations:** 1 Programa de Investigación en Enfermedades Tropicales, Escuela de Medicina Veterinaria, Universidad Nacional, Heredia, Costa Rica; 2 Centro de Investigación en Enfermedades Tropicales, Universidad de Costa Rica, San José, Costa Rica; 3 Centre d'Immunologie de Marseille-Luminy (CIML), Aix-Marseille University, UM2, Marseille, France; 4 Institut National de la Santé et de la Recherche Médicale (INSERM), U1104, Marseille, France; 5 Centre National de la Recherche Scientifique (CNRS), UMR7280, Marseille, France; 6 Department of Cell Microbiology, Max Planck Institute for Infection Biology, Berlin, Germany; 7 Biological Sciences, Inflammation Research Network, University of Calgary, Calgary, Alberta, Canada; 8 Instituto Clodomiro Picado, Facultad de Microbiología, Universidad de Costa Rica, San José, Costa Rica; University of California, Davis, UNITED STATES

## Abstract

Most bacterial infections induce the activation of polymorphonuclear neutrophils (PMNs), enhance their microbicidal function, and promote the survival of these leukocytes for protracted periods of time. *Brucella abortus* is a stealthy pathogen that evades innate immunity, barely activates PMNs, and resists the killing mechanisms of these phagocytes. Intriguing clinical signs observed during brucellosis are the low numbers of *Brucella* infected PMNs in the target organs and neutropenia in a proportion of the patients; features that deserve further attention. Here we demonstrate that *B*. *abortus* prematurely kills human PMNs in a dose-dependent and cell-specific manner. Death of PMNs is concomitant with the intracellular *Brucella* lipopolysaccharide (*Br*-LPS) release within vacuoles. This molecule and its lipid A reproduce the premature cell death of PMNs, a phenomenon associated to the low production of proinflammatory cytokines. Blocking of CD14 but not TLR4 prevents the *Br*-LPS-induced cell death. The PMNs cell death departs from necrosis, NETosis and classical apoptosis. The mechanism of PMN cell death is linked to the activation of NADPH-oxidase and a modest but steadily increase of ROS mediators. These effectors generate DNA damage, recruitments of check point kinase 1, caspases 5 and to minor extent of caspase 4, RIP1 and Ca^++^ release. The production of IL-1β by PMNs was barely stimulated by *B*. *abortus* infection or *Br*-LPS treatment. Likewise, inhibition of caspase 1 did not hamper the *Br*-LPS induced PMN cell death, suggesting that the inflammasome pathway was not involved. Although activation of caspases 8 and 9 was observed, they did not seem to participate in the initial triggering mechanisms, since inhibition of these caspases scarcely blocked PMN cell death. These findings suggest a mechanism for neutropenia in chronic brucellosis and reveal a novel *Brucella*-host cross-talk through which *B*. *abortus* is able to hinder the innate function of PMN.

## Author Summary

The absence of obvious clinical symptoms during the early stages of brucellosis is linked to the *Brucella* stealthy strategy and its non-canonical PAMPs, which are low PRRs agonists. Still, there are clinical profiles that require explanation. For instance ‒despite the fact that neutrophils readily ingest *Brucella* during the onset of infection, brucellosis courses without neutrophilia, and just a low number of infected neutrophils are present in target organs. In the chronic phases, a significant proportion of the patients display absolute neutropenia and bone marrow pancytopenia linked to the myeloid cell linage. Examination of the *Brucella* infected bone marrow reveals granulomas and phagocytosis of myeloid cells. Based on these observations we explored the fate of native neutrophils during their interaction with *Brucella*. We found that the bacterium induces the premature cell death of neutrophils without inducing proinflammatory phenotypic changes. This event was reproduced by the lipid A of the *Brucella* LPS and depends on NADPH-oxidase activation and low ROS formation. We believe that this phenomenon explains ‒at least in part‒ the hematological and histological profiles observed during brucellosis. In addition, it may be that dying *Brucella*-infected neutrophils serve as “Trojan horse” vehicles for infecting phagocytic cells without promoting activation.

## Introduction

Polymorphonuclear leukocytes (PMNs) represent a key cellular component of the host’s antibacterial arsenal. Once in the circulation, the average lifespan of PMNs is close to 5.4 days, period after which they undergo spontaneous apoptosis [1]. This is in frank contrast to the previously reported short lifespans of a few hours for these cells [2]. Then, these dead PMNs are removed by phagocytic cells laying in the reticuloendothelial system, such as monocytes (Mo), macrophages (Mϕ) and dendritic cells (DCs) [3]. This physiological phenomenon does not induce proinflammatory signals and is regarded as a constitutive mechanism to maintain leukocyte homeostasis [4].

Upon bacterial infection, PMNs are activated and migrate into tissues, where they may survive three to five days to perform their phagocytic, microbicidal and proinflammatory functions [1,5]. These events are part of the innate immune response commonly triggered by pathogen-associated molecular patterns (PAMPs) [6] or by danger signals that guide the PMNs response [7].

A variety of microbes have evolved strategies to influence the timing and mode of PMN cell death [8–10]. For instance, *Shigella flexneri* kills PMNs by necrosis, a process characterized by the release of tissue-injurious granular proteins. This contributes to disruption of the intestinal epithelial barrier, leading to the dysentery observed in shigellosis and allowing the bacterium to enter its colonic host cells [11]. Similarly, *Pseudomonas aeruginosa* infections may cause lysis or oncosis of PMNs, leading to persistent infections by depleting these cells and contributing to the pulmonary pathophysiology by facilitating bacterial extracellular replication [12,13]. Others, such as the obligate intracellular *Anaplasma phagocytophilum* and *Chlamydia pneumoniae* are able to inhibit PMN cell death to achieve intracellular replication within these leukocytes [14,15].

*Brucella* microorganisms are stealthy alpha-protobacterial intracellular pathogens of mammals, including humans [16,17]. In the early stages of infection, *Brucella* minimizes the host proinflammatory response, opening an immunological window that allows this bacterium to invade and reach sheltered intracellular niches before adaptive immunity becomes effective [16,18,19]. Once established, *Brucella* organisms survive and extensively replicate within the intracellular milieu of Mo, Mϕ, DCs and placental trophoblasts [20,21]. As part of its parasitic strategy, *Brucella* inhibits apoptosis and prolongs the life of these infected mononuclear phagocytic cells [16,22]. Although *Brucella* is readily internalized by PMNs [23,24], the bacterium survives inside the phagosomes of these cells resisting their killing action including oxidative components and isolated lysosomal extracts [16,25,26].

During the course of human and animal brucellosis, there are several clinical and pathological features related to PMNs which biological mechanisms remain unclear. Among the most striking signs are the neutropenia observed during chronic brucellosis, the absence of recruitment of PMNs at the site of infection and the low numbers of *Brucella* infected PMNs in the target organs [16,27–30]. Moreover, PMNs have an unexpected influence in dampening the immune response against intracellular *Brucella* infection and strengthen the notion that PMNs actively participate in regulatory circuits shaping both innate and adaptive immunity [19].

In an attempt to improve our understanding of the mechanisms underlying the fate of PMNs during brucellosis, we have explored the outcome of these leukocytes upon interaction with *Brucella abortus*. Our findings reveal a novel microbial-host cross-talk through which *B*. *abortus* is able to hinder and evade host innate PMN response and suggest a mechanism by which *Brucella* may hamper the presence of infected PMNs in the target organs and promote neutropenia during chronic brucellosis.

## Results

### *B*. *abortus* resists the killing action of PMNs

**Fig 1:**
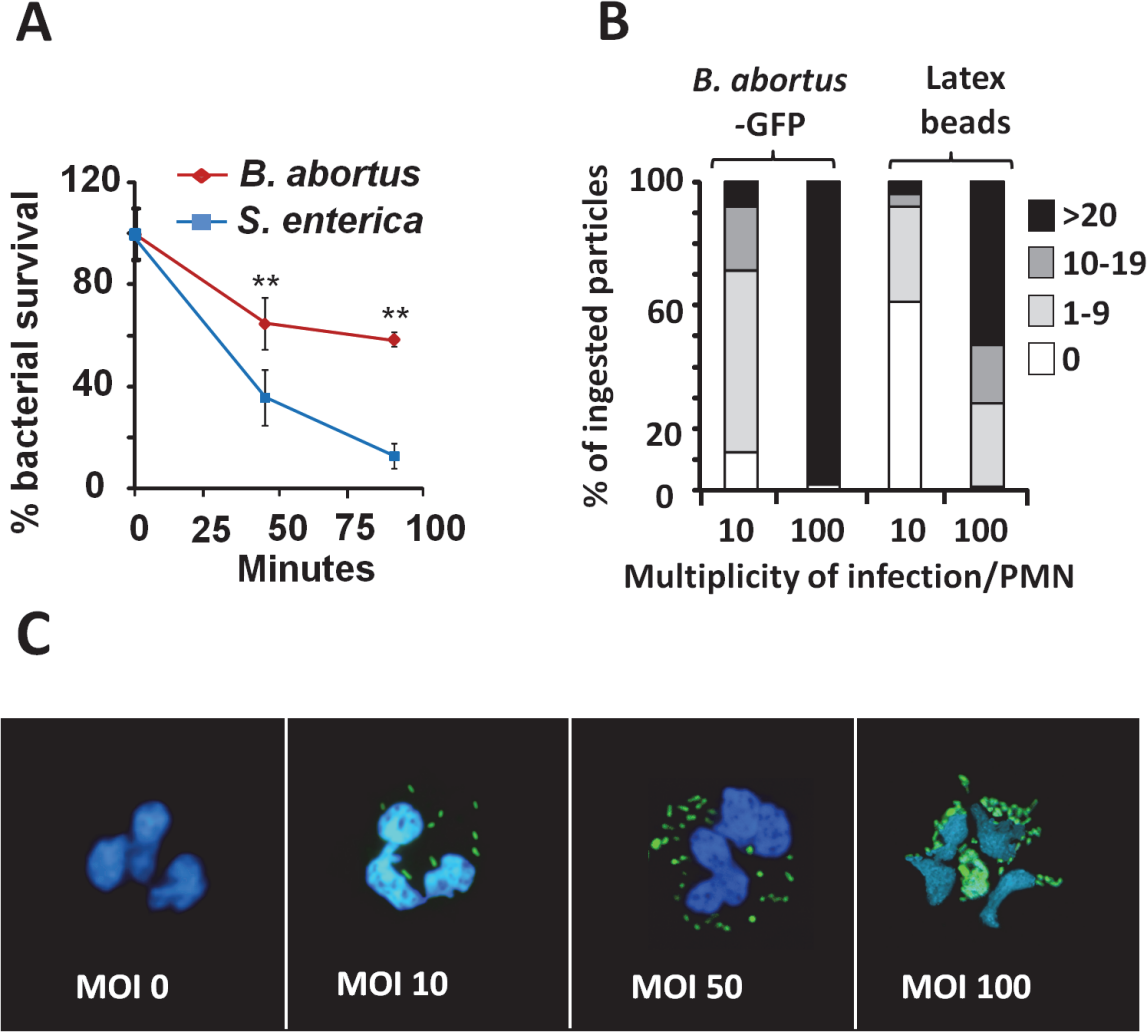
*B*. *abortus* is partially resistant to the killing action of PMNs. (A) PMNs were isolated from blood and incubated with *B*. *abortus* or *S*. *enterica* (MOI 5) and CFUs determined at different time points. (B) Heparinized blood was incubated with *B*. *abortus*-GFP or fluorescent latex beads for two hours (MOI 10 or 100). Blood smears were then fixed and mounted with ProLong Gold Antifade Reagent with DAPI. At least 100 PMNs were counted per sample and the number of intracellular bacterial or latex particles determined in each PMN and the proportion expressed as % of phagocytized particles. (C) Human PMNs infected with different MOI of *B*. *abortus*-GFP and stained as in “B”. Microscope images are at 400 × magnification. Representative PMNs with DAPI-stained nuclei and intracellular green fluorescent *B*. *abortus* were photographed under the microscope using the appropriate color filter channel. Images were cut from microscope field, contrasted and saturated using Hue tool to obtain suitable color separation. Images were then merged using Adobe Photoshop 8 software. Experiments were repeated at least three times. Values of *p*<0.01 (**) are indicated.

Confirming previous reports [16,18,31], *Brucella* is more resistant than other bacteria to the killing action of PMNs (Fig 1A). This resistance is not related to reduced bacterial internalization, since at multiplicity of infection (MOI) of 5, both *B*. *abortus* and *Salmonella enterica*, were phagocytized at similar rates. Due to the toxic effects mediated by *Salmonella* on PMNs, higher MOIs of this bacterium were precluded. Compared to latex beads, fluorescent *B*. *abortus-*GFP was internalized more efficiently by PMNs at different MOIs, suggesting an active PAMP receptor-mediated phagocytosis (Fig 1B). Early phagocytosis of *B*. *abortus*-GFP (MOI < 50) was not accompanied by obvious PMN shape changes such as nuclear rounding, chromatin condensation, cell fragmentation, degranulation (Fig 1C) or myeloperoxidase activity [16]. This observation is in agreement with previous reports [16,25,31,32]. Only when high loads of *B*. *abortus*-GFP were tested (MOI > 50) changes in nuclear morphology was detected in a proportion of PMNs containing more than 50 bacteria/cell (Fig 1C).

### *B*. *abortus* infection induces PMN cell death in a dose-dependent manner

**Fig 2:**
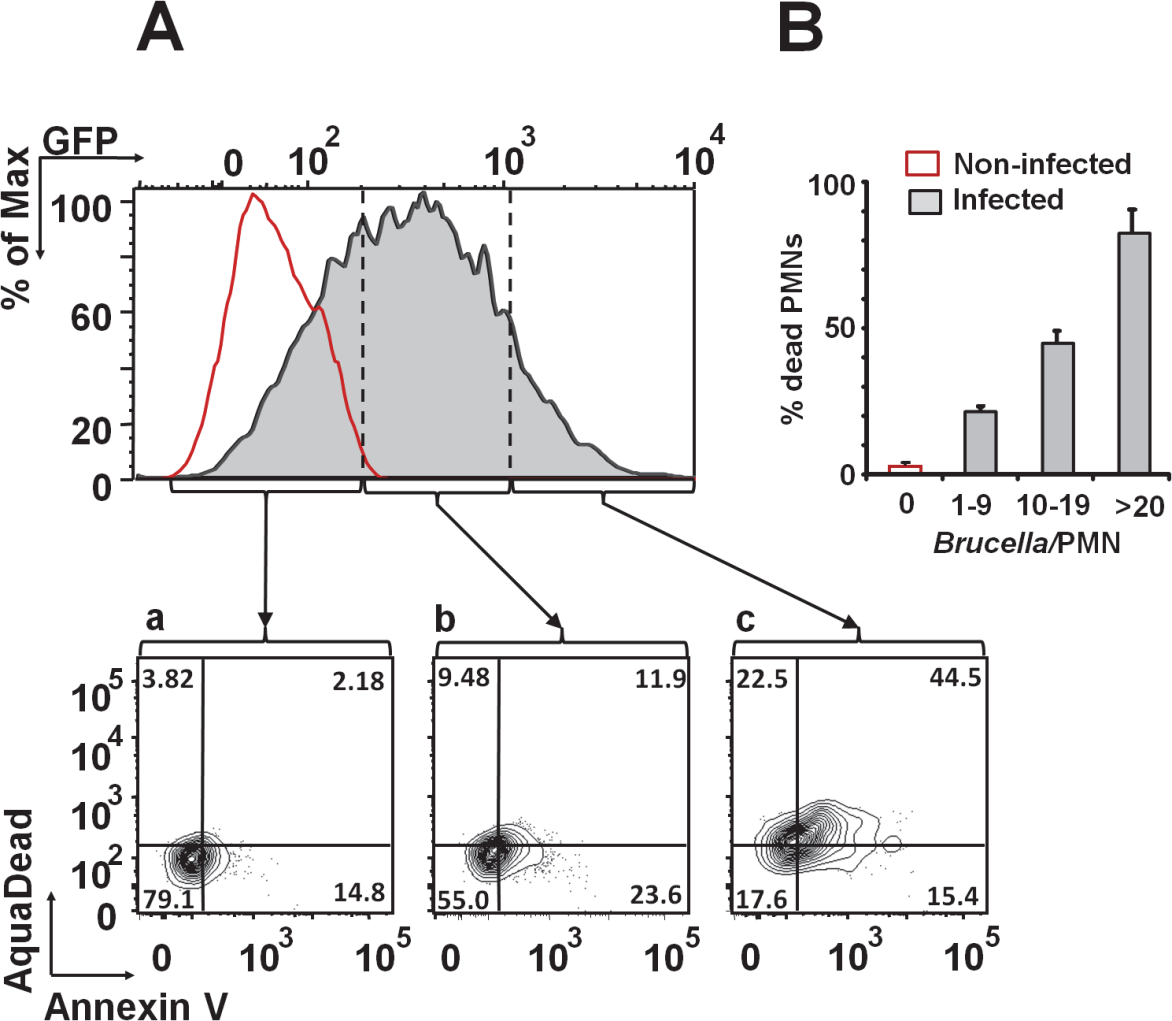
*B*. *abortus* infection induces PMN cell death in a dose dependent manner. (A) Heparinized blood was incubated with *B*. *abortus*-GFP (MOI 10) for two hours and PMNs population analyzed for cell death using AquaDead and Annexin V markers. GFP fluorescence intensity was used to differentiate among three categories: (a) low, (b) intermediate and (c) high infection. (B) Percentages of PMNs positive for any marker in relation to the number of internalized bacteria are shown. Experiments were repeated at least three times.

**Fig 3:**
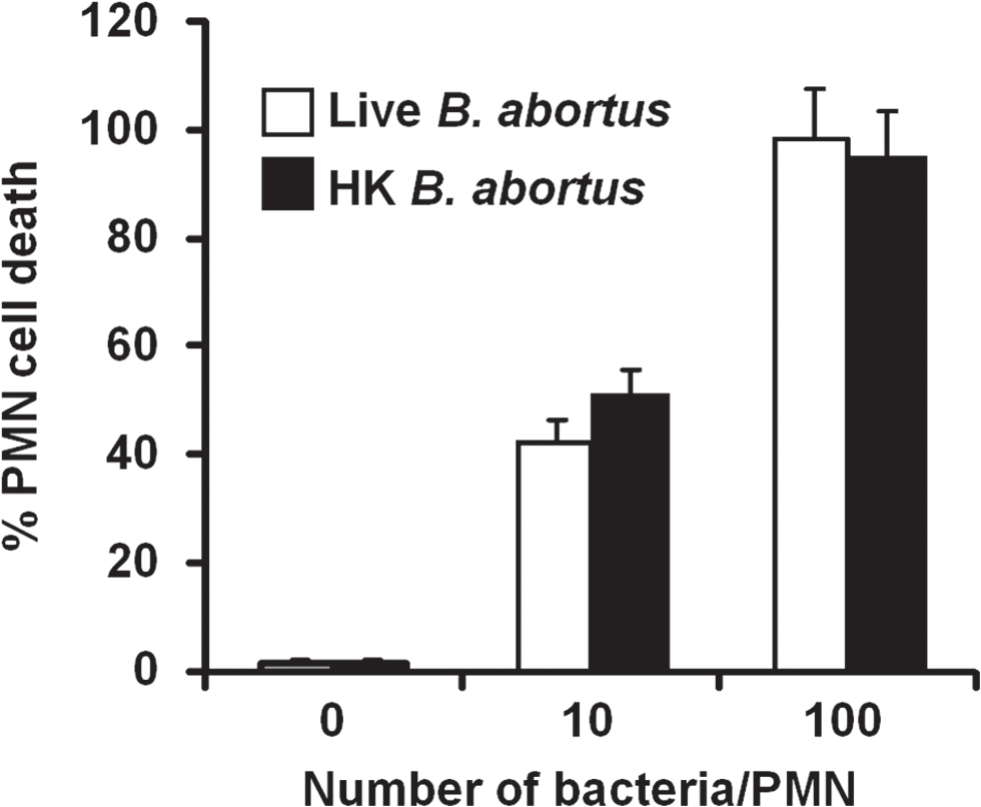
Live and heat-killed *B*. *abortus* induce PMN cell death. Heparinized blood was incubated with live or heat-killed (HK) *B*. *abortus* for two hours (10 and 100 bacteria/PMN). PMN population was analyzed by flow cytometry for cell death using AquaDead and Annexin V markers, as described in Fig 2. Percentages of PMNs positive for any marker were determined. Experiments were repeated at least three times. No significant differences were detected between live and HK *B*. *abortus*.

After infection with *Brucella*-GFP, PMN cell death was assessed by flow cytometry using Annexin V and AquaDead as markers. After two hours of incubation (MOI = 10), *Brucella* infected PMNs (whole blood or purified PMNs, see below) became positive for both markers, following a bacterial dose dependence (Fig 2). This phenomenon did not require live bacteria, since similar effects were observed in PMNs exposed to equivalent doses of live or heat killed *B*. *abortus* (HKBA) (Fig 3).

### *B*. *abortus* releases *Br-*LPS inside vacuoles of PMNs

**Fig 4:**
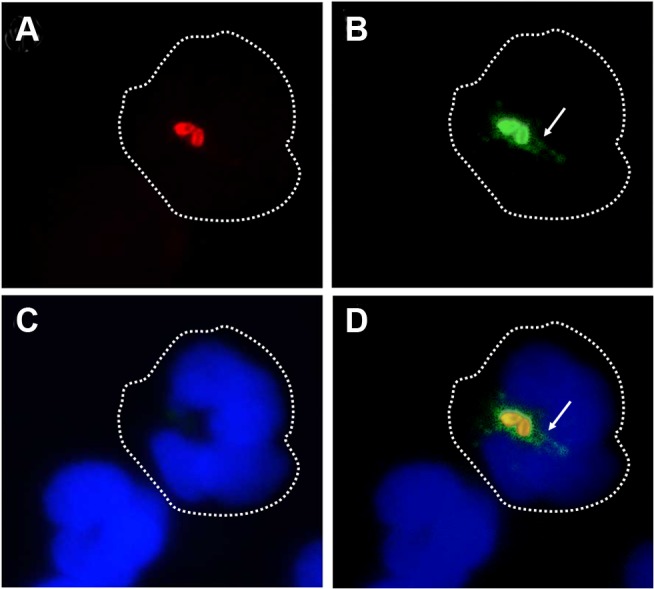
*Br*-LPS is released inside PMNs. Heparinized blood was incubated with *B*. *abortus-*RFP for one hour (MOI 2). Blood smears were fixed, stained with anti-*Brucella* LPS FITC (green) and mounted with ProLong Gold Antifade Reagent with DAPI. (a) *B*. *abortus-*RFP, (b) IgG-FITC anti-*Brucella* LPS staining, (c) PMN DAPI staining and (d) merged images. Shed *Brucella* LPS (white arrow) is pointed. Representative PMNs with DAPI-stained nuclei and intracellular *B*. *abortus* were photographed under the microscope using the appropriate color filter channel. Images were cut from microscope field, contrasted and saturated using Hue tool to obtain suitable color separation. Images were then merged using Adobe Photoshop 8 program. Microscope images are at 1000 × magnification.

We have demonstrated that *B*. *abortus* sheds non-toxic *Br*-LPS inside cells and that this molecule traffics in vacuoles and influences antigen presentation to T cells [33–35]. Following this, we explored the shedding of *Br-*LPS inside PMNs by live *B*. *abortus*. For this purpose, we used a double labeling fluorescence method [36]. First PMNs were infected with *B*. *abortus-*RFP at a MOI of 5. After 1 h of incubation, PMNs were permeabilized and treated with anti-*Br-*LPS FITC-antibody and counterstained with DAPI. This approach revealed that significant amounts of *Br-*LPS molecules (green fluorescence) were released intracellularly by live (red fluorescent) *Brucella* in the proximity of bacteria-containing PMN phagosomes (Fig 4). Almost all *B*. *abortus-*RFP infected PMNs exhibited this pattern after 1 h infection, most strikingly evident by immunofluorescence in cells containing between 2–3 bacteria/PMN.

**Fig 5:**
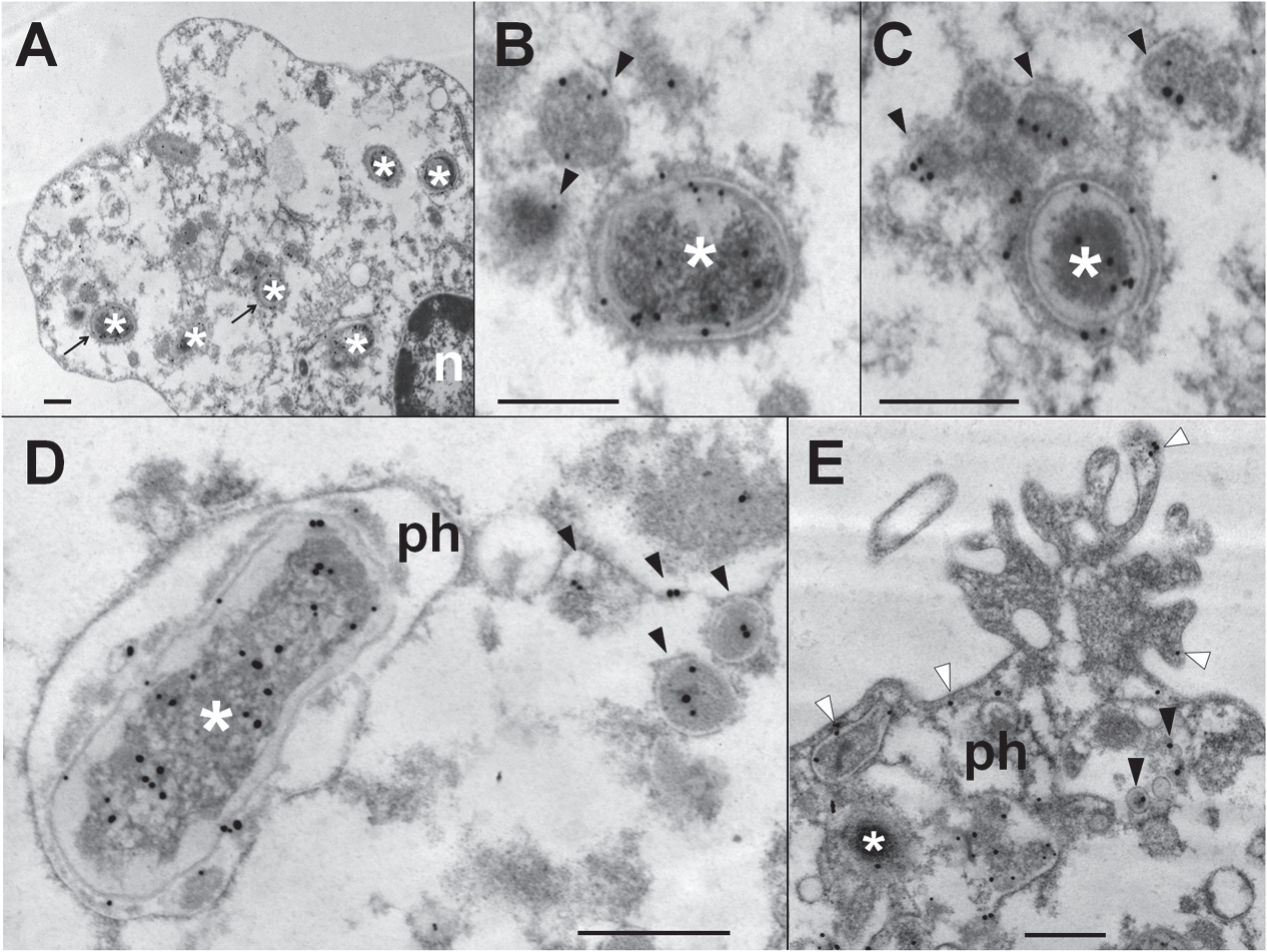
*Br-*LPS released inside cells is mostly found within vacuolar compartments of PMNs. Purified human PMNs 5 ×10^6^ were infected with *B*. *abortus* 2308 at MOI 20. After one hour incubation, infected cells were fixed and processed for immunogold staining and electron microscopy. Detection of *Br*-LPS was performed using mouse IgG anti Br-LPS in combination with protein-A/protein-G colloidal gold 15 nm. (A) PMN (n, nucleus of cell) with intracellular *B*. *abortus* (white asterisk) and immunogold detection of *Br*-LPS. (B) and (C) correspond to amplified sections pointed with arrows from “A” panel; *B*. *abortus* (white asterisk) and immunogold detection of *Br*-LPS inside vacuoles (pointed by black arrow heads). (D) *B*. *abortus* (white asterisk) within a phagosome (ph) and vacuoles containing immunogold labeled *Br*-LPS (black arrow heads). (E) PMN membrane ruffle showing immunogold detection of *Br*-LPS associated to the membrane (white arrow heads) and *B*. *abortus* (white asterisk) debris inside a phagosome (ph) and immunogold detection of *Br*-LPS inside vacuoles (black arrow heads). No colloidal gold particles were observed when IgG purified from normal mouse serum was used for controlling the specificity of the reaction. Bar represents 500 nm.

In order to determine if *Br*-LPS was released inside vacuoles or translocated to the cytosol, *B*. *abortus* infected PMNs were subjected to immunodetection of *Br*-LPS by electron microscopy. Regular osmium tetroxide staining of infected PMNs (1 hour) demonstrated that all phagocytized *B*. *abortus* reside inside phagosomes, and just a few of them within phagolysosomes, confirming previous results [37]. As expected, sensitive staining for detection of immunogold particles revealed the presence of *Br-*LPS in the bacterial cells. However, vesicles in the proximity of the ingested *Brucella* also contained gold particles, indicating the presence of *Br*-LPS within vacuoles (Fig 5). In some cells, immunogold stained *Br*-LPS molecules were detected within a phagosome containing partially digested bacteria or in the proximity of cell membrane ruffle-like structures (Fig 5E). Gold particles were practically absent in the cytosol and not detected in the extracellular milieu (Fig 5A).

### *Br*-LPS specifically induces the cell death of PMNs

**Fig 6:**
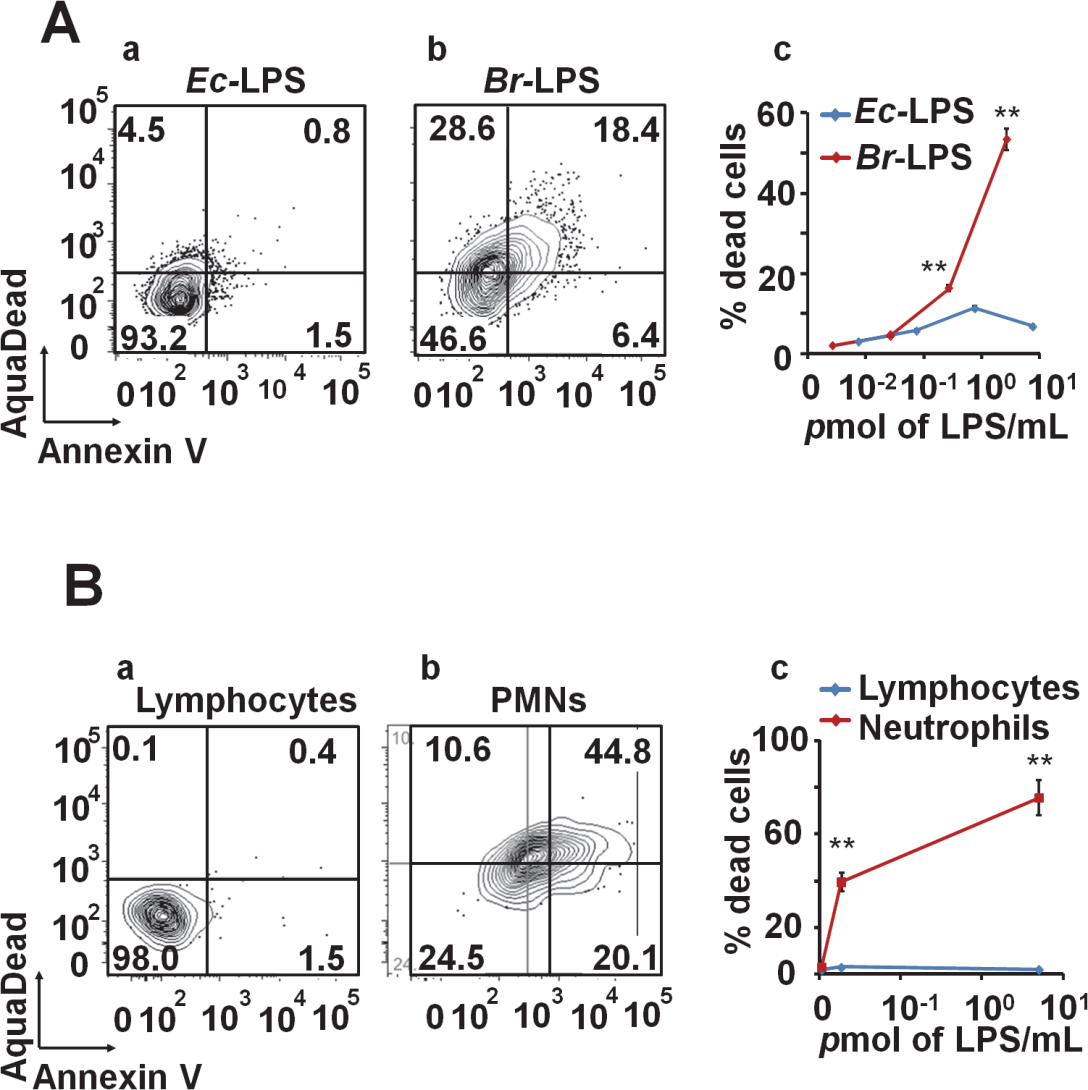
*Br-*LPS induces cell death of PMN in a dose dependent manner. (A) Heparinized blood was incubated with 100μg/mL of (a) *Ec-*LPS (corresponding to 7.5 pmol/mL) or (b) *Br-*LPS (corresponding to 3 pmol/mL) for two hours and the PMN population was analyzed by AquaDead and Annexin V markers as in Fig 2. (c) Percentages of PMNs positive for any marker treated and with various concentrations of LPS are shown. (B) Human blood was incubated with 3 pmol/mL of *Br-*LPS for two hours. (a) Lymphocyte and (b) PMN populations were analyzed by AquaDead and Annexin V markers. (c) Percentages of lymphocytes and PMNs positive for any marker treated and with various concentrations of *Br-*LPS for are shown. Experiments were repeated at least three times. Values of *p*<0.01 (**) are indicated.

Intracellular *Br*-LPS influences the antigen presentation of Mϕ without affecting the survival of these cells [34,35]. Therefore, we assessed the effects of *Br*-LPS on PMNs cell survival. As demonstrated in Fig 6A, *Br*-LPS induced PMN cell death in a dose-dependent manner in blood or in purified (see the results presented in the next sections) PMNs. This effect was specific for PMNs since other cells, such as lymphocytes, treated and gated under the same conditions, did not display death cell markers (Fig 6B). Consistent with previous observations [22,38], *Br*-LPS did not induce cell death of Mϕ, Mo and DCs. In contrast, *Escherichia coli* LPS (*Ec*-LPS) did not induce cell death in blood (Fig 6A) or in purified PMNs under the same experimental conditions.

**Fig 7:**
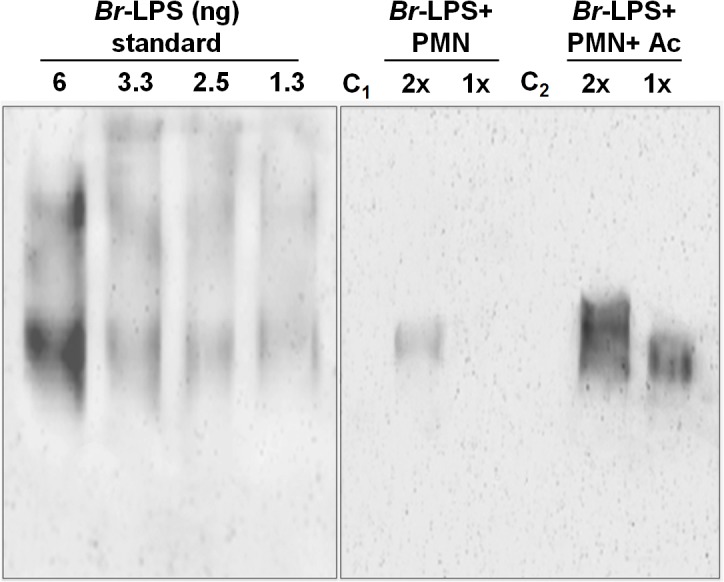
Quantities of *Br*-LPS interacting with PMNs. The quantities of *Br*-LPS associated to PMNs were determined by Western blotting using a monoclonal antibody against *Br*-LPS conjugated with peroxidase enzyme. All wells were loaded with 15 μL of the respective preparation. The amounts of purified *Br*-LPS in the left panel were used to estimate the quantities based on a standard curve ranging from 0.06 ng to 12 ng (only wells from 1.3–6 ng are shown). The right panel corresponds to the assay: purified PMNs were incubated with *Br*-LPS and the associated amounts determined by Western blot (Br-LPS+PMNs). In order to have a saturating positive control, the assay was also performed in the presence of human antibodies against *Br*-LPS (*Br*-LPS+PMNs+Ac). Controls included the assay performed with *Br*-LPS in the absence (C_1_) or presence of human antibodies (C_2_) but in the absence of PMNs. PMNs alone did not show any signal. Notice that the *Br*-LPS molecules associated to PMNs corresponded to the lower molecular weight fraction (~30–40 MW). The amounts of *Br*-LPS were estimated to be in the range of 5–25 ng/10^6^ PMNs, corresponding to less than 0.25% of the original *Br*-LPS added. The amounts of associated *Br*-LPS in the presence of antibodies were between 10–50 ng/10^6^ PMNs. These estimated quantities were from four different experiments. The read-out of the corresponding bands was performed by densitometry.

In view of the relatively high amounts of *Br*-LPS added to induce PMN cell death, a quantitative determination of the *Br*-LPS interacting with these cells was performed. For this purpose, purified PMNs were incubated with *Br*-LPS and the associated amounts determined by Western blotting (Fig 7). In order to have a saturating positive control, the assay was also performed in the presence of human antibodies against *Br*-LPS. The estimated quantities of associated *Br*-LPS in the absence of antibodies ranged between 5–25 ng/10^6^ PMNs. Likewise, the amounts of associated *Br*-LPS in the presence of antibodies were between 10–50 ng/10^6^ PMNs. This result indicates that the actual quantities of *Br*-LPS interacting with PMNs under these experimental conditions corresponded just 0.05–0.25% of the total *Br*-LPS added. As expected, antibodies increased close to 10 times the quantities of *Br*-LPS associated to PMNs through the concourse of Fc receptors. It should be noticed that the molecules associated to PMNs corresponded to the lower molecular weight (~30–40 MW) fraction of *Br*-LPS. This indicates that among all the *Br*-LPS molecules available, just specific classes are selected by PMNs.

### The lipid A moiety of the *Br-*LPS is responsible for the induction of PMN cell death

**Fig 8:**
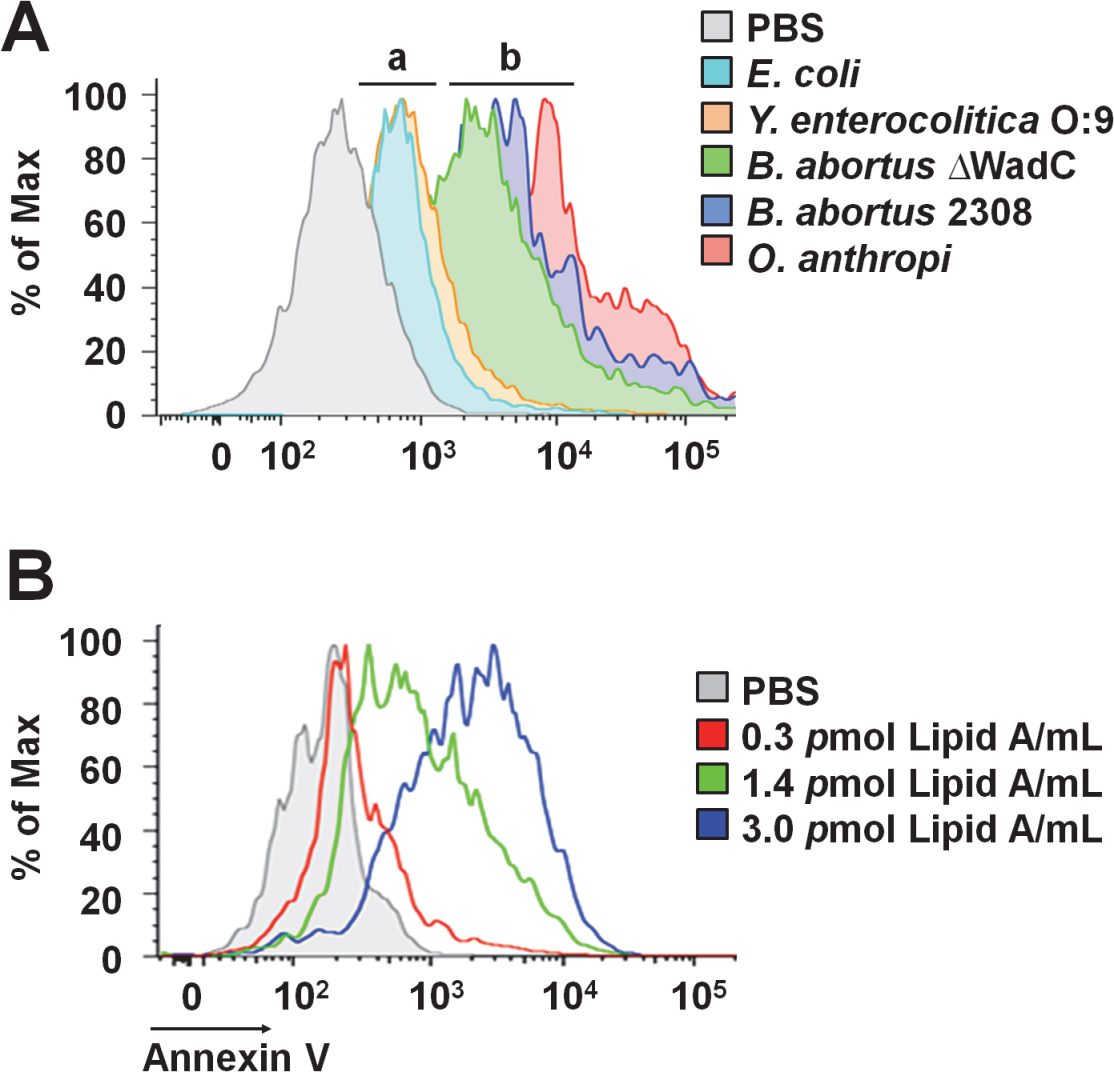
*Brucella* lipid A induces PMNs cell death in a dose dependent manner. (A) Heparinized blood was incubated for two hours with LPSs of *Y*. *enterocolitica* O:9 (3 pmol/mL), of *E*. *coli* (7.5 pmol/mL), of *B*. *abortus* 2308 (3 pmol/mL), of *B*. *abortus* ∆WadC (3 pmol/mL) and of *O*. *anthropi* (2 pmol/mL), all corresponding to 100μg/mL of LPS. The LPSs differed in at least one of the moieties (O-chain, core and lipid A) with *B*. *abortus* 2308 LPS: (a) LPSs possessing lipid As that differ from *B*. *abortus* 2308 LPS, (b) LPSs possessing lipid As structures similar to *B*. *abortus* 2308 LPS. (B) Heparinized blood treated with different concentrations of *B*. *abortus* 2308 lipid A for two hours. In all assays, PMN population was gated and analyzed by Annexin V marker and the geometric means of histograms displayed as relative units. Experiments were repeated at least three times.

The non-toxic *Br-*LPS is built of an O-chain constructed of N-formyl perosamine sugar homopolymer, a positively charged core oligosaccharide and a lipid A containing a diaminoglucose disaccharide backbone substituted with long chain hydroxylated, cyclic and non-hydroxylated fatty acids (S1 Fig). In an attempt to identify the moiety responsible for inducing the PMN cell death, we first tested the biological action of different LPSs that shared at least some of the *Br-*LPS structural features [18,39–45]: i) *Yersinia enterocolitica* O:9 LPS displays the same O-chain homopolymer as *Br-*LPS but has different lipid A and core oligosaccharide; ii) *Ochrobactrum anthropi* LPS shares the lipid A structural features with *Br-*LPS but possesses different O chain and core oligosaccharide; iii) *B*. *abortus* ∆WadC LPS displays the same lipid A and O chain as the *Br-*LPS, but has a different core oligosaccharide; finally, iv) the overall structure of *Ec-*LPS differs from that of *Br-*LPS, but it shares the lipid A and core features with *Y*. *enterocolitica* O:9 LPS. As shown in Fig 8A, LPSs from *B*. *abortus* ∆Wad*C* and *O*. *anthropi* sharing the same lipid A structure as the *Br*-LPS were able to induce cell death more readily than other LPSs. A similar pattern of PMN cell death was observed when these cells were treated with increasing quantities of purified *B*. *abortus* 2308 lipid A (Fig 8B). Altogether, these results demonstrate that the lipid A of *Br*-LPS is the moiety responsible for inducing the premature cell death of human PMNs.

### Blocking of CD14 molecule prevents the *Br-*LPS-induced PMN cell death

It is well known that the coordinated interaction of CD14, MD-2/TLR4 molecules mediate LPS recognition in mammalian cells [46] and that binding of these membrane molecules may promote cell survival or cell death depending on the context [47,48]. Therefore, we explored the roles of TLR4 and CD14 *Br*-LPS-induced the cell death in PMNs.

**Fig 9:**
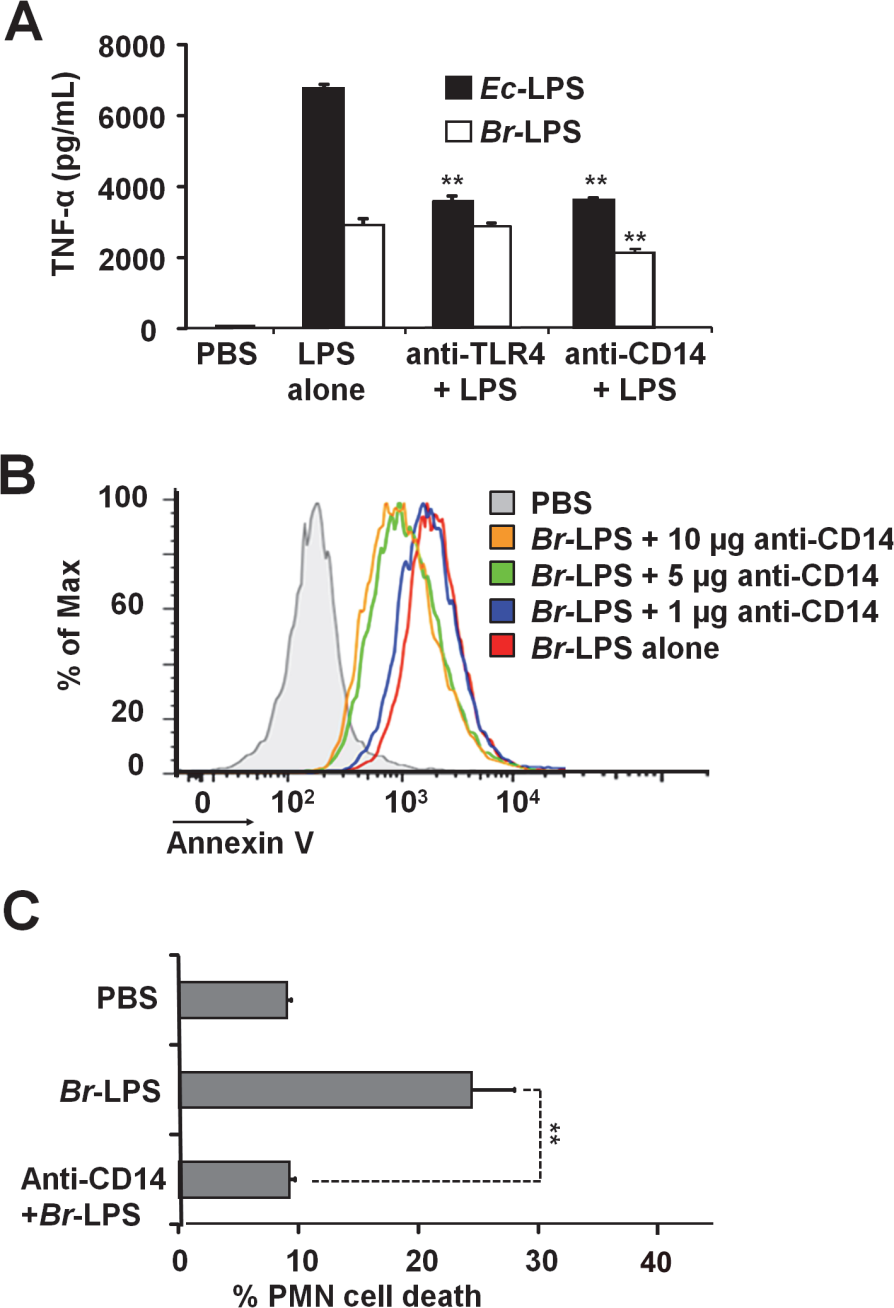
Neutralization of CD14 protects against *Br-*LPS-induced PMN cell death. (A) Heparinized blood was incubated for two hours with 0.4 pmol/mL of *Ec-*LPS or 3 pmol/mL of *Br-*LPS. Prior to LPS stimulation, some samples were previously treated with anti-TLR4 (1 μg/mL) or anti-CD14 (5μg/mL) antibodies and TNF-α secretion quantified in plasma by ELISA. Values of *p*<0.01 (**) are indicated in relation to their respective LPS control. (B) Heparinized blood was incubated with *Br-*LPS (3 pmol/mL) alone or previously neutralized with different quantities of anti-CD14. PMN population was gated and analyzed by Annexin V marker. Geometric means of histograms displayed as relative units. Experiments were repeated at least three times. (C) Purified PMNs were incubated for two hours with *Br-*LPS (1.5 pmol/mL). Prior to LPS stimulation, some samples were previously treated with anti-CD14 (5μg/mL) antibodies and PMN population gated and analyzed by AquaDead marker. Anti-CD14 alone does not have any significant effect in PMN cell death. Value of *p*<0.01 (**) is indicated in relation to the *Br*-LPS control.

When TLR4 or CD14 receptors were blocked with specific antibodies prior to the exposure of blood with *Ec-*LPS, the secretion of TNF-α was significantly abrogated (Fig 9A), indicating that the amounts of antibodies used were suitable. Despite of the lower amounts of TNF-α induced by *Br*-LPS as compared to those stimulated by *Ec*-LPS, the blocking of TLR4 does not have any effect on the action of the former bacterial molecule on blood cells. This phenomenon is consistent with previous findings demonstrating that *Br-*LPS is a poor agonist of the MD-2/TLR4 pathway [16,43]. Likewise, when TLR4 was blocked, PMN cell death mediated by *Br-*LPS was not inhibited (S2 Fig). In contrast, anti-CD14 antibodies significantly inhibited the secretion of TNF-α (Fig 9A) in blood as well as PMN cell death induced by *Br-*LPS (Fig 9B). Since anti-CD14 treatment of blood could modulate other leukocytes and influence the dead of PMNs, we then performed the experiment using purified PMNs (Fig 9C) to confirm the blocking effect of anti-CD14. In preparations of purified human PMNs, blocking of CD14 totally abolished the *Br*-LPS induction of cell death after a short incubation (Fig 9C). Anti-CD14 alone or low amounts of this antibody (≤1μg) did not have any observable effect in PMN cell death.

### *Br-*LPS-induced PMN cell death correlates with low ROS formation

**Fig 10:**
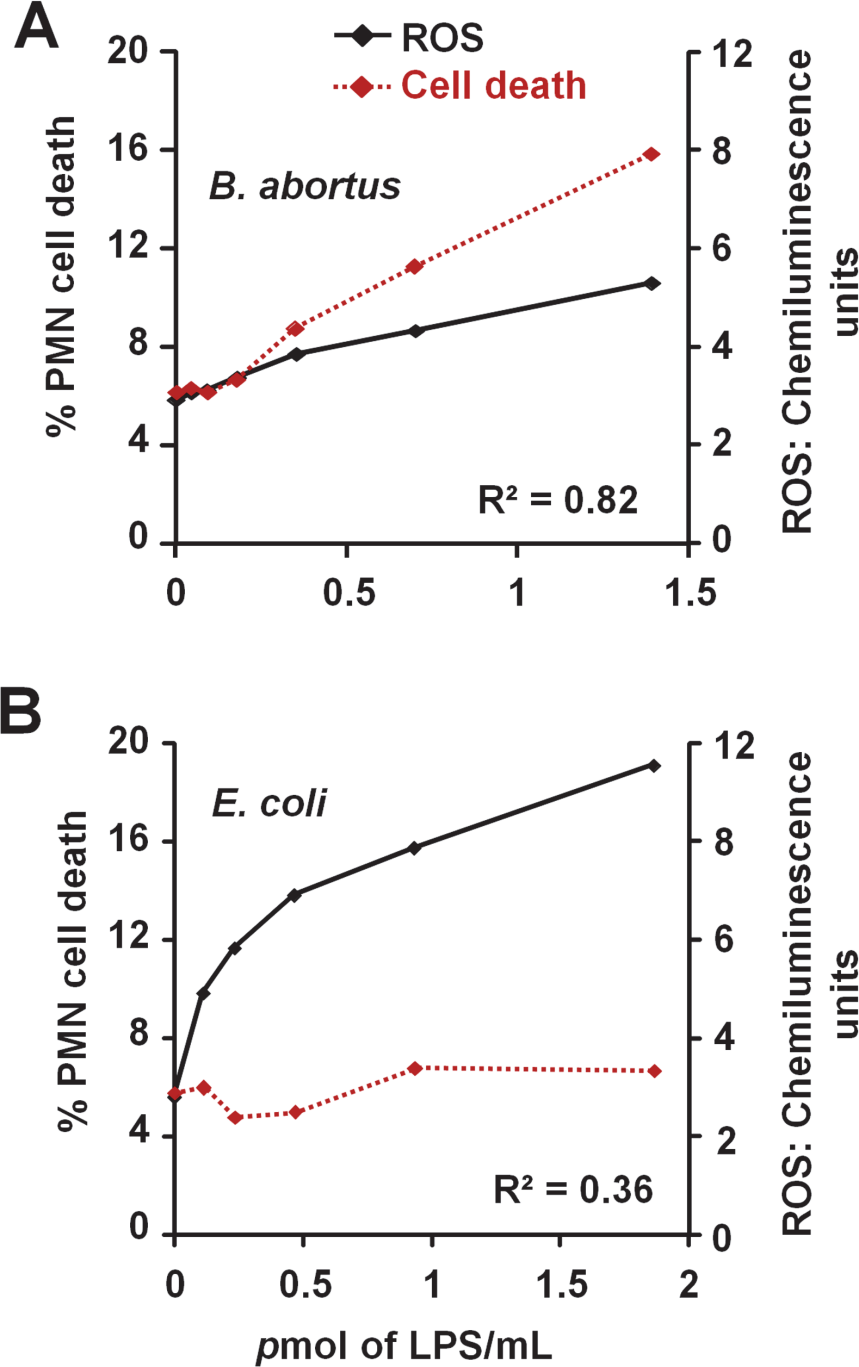
*Br*-LPS-induced PMN cell death correlates with low ROS formation. Purified human PMNs were seeded on serum-uncoated plates and treated with various concentrations of *Br-*LPS or *Ec-*LPS for 6.5 h. (A) ROS kinetics production was monitored for 90 minutes by luminol-amplified chemiluminescence and the maximum obtained value for each LPS concentration plotted (black line). (B) Cell death of purified PMNs was monitored by evaluation of Sytox green fluorescence (shown as percentage of cell death relative to PMA-induced cell death) (red dotted line). Figure represents the outcome of a single experiment. Similar results were obtained in repeated experiments. Correlation *R* ^*2*^ was obtained by using the Excel tool facility.

The low and slow kinetics of ROS formation induced by *Br*-LPS correlates with the kinetics of the PMN cell death observed (Fig 10A). Although it is dose dependent, this profile is in clear contrast and opposite to the kinetics of ROS formation and cell death measured in *Ec-*LPS treated PMNs (Fig 10B). *Brucella*-infected PMNs did not undergo NETosis or display typical signs of apoptosis or necrosis (Fig 1C). Likewise, the doses of *Br*-LPS that promoted PMN cell death failed to induce NETosis (S4 Fig) or degranulation, as demonstrated before [49]. Therefore, this phenomenon seems to be specifically mediated by *Br-*LPS and its lipid A. This also agrees with previous data demonstrating that these bacterial molecules barely induce degranulation or activation of PMNs [49].

### *Br*-LPS triggers PMN cell death through the action of NADPH-oxidase and ROS mediators

**Fig 11:**
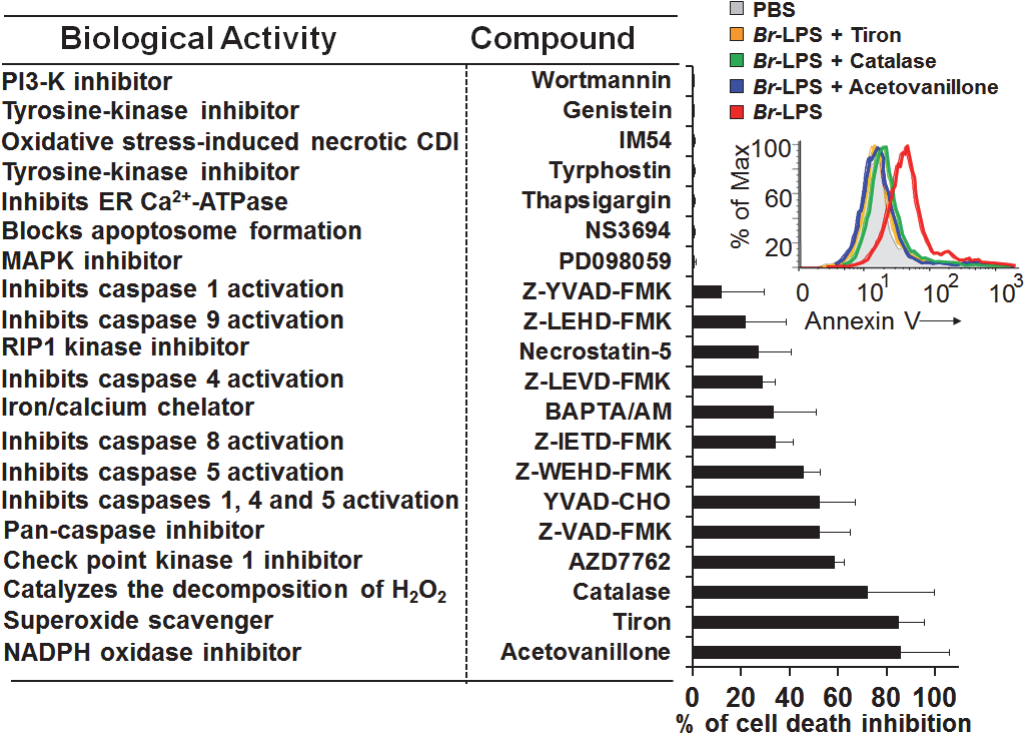
Inhibitory action of various compounds on the *Br*-LPS-induced PMN cell death. Prior to *Br*-LPS stimulation, samples were treated with wortmanin (50 nM), genistein (100 μM), IM-54 (10 μM), tyrphostin (250 μM), thapsigargin (50 nM), NS3694 (10 μM), PD098059 (50 μM), Z-YVAD-FMK (10 μM), Z-LEHD-FMK (10 μM), necrostatin-5 (10 μM), Z-LEVD-FMK (10 μM), BAPTA/AM (10 μM), Z-IETD-FMK (10 μM), Z-WEHD-FMK (10 μM), YVAD-CHO (50 μM), Z-VAD-FMK (10 μg/mL), AZD7762 (30 μM), catalase (2800 U/mL), tiron (2 mg/mL), acetovanillone (100 μg/mL) or PBS. After treatment with the inhibitory compounds, samples were incubated with *Br*-LPS (1.5 pmol/mL) for 2 hours. Samples were further processed and analyzed by cytometry for cell death with Annexin V as described above. Geometric means of histograms displayed as relative units. In the upright corner, the read out procedure of the inhibitory action of tiron, catalase and acetovanillone is presented. Values were estimated as relative units of the geometric means of histograms. Each experiment was repeated at least three times.

Many of the cell death features displayed by PMNs are unique for these leukocytes [50–52]. Since microscopically the *Brucella*-induced PMN cell death does not fit with any of the classical cell death types described for these phagocytes, then we investigated the action of several chemical inhibitors (Fig 11). Among the most conspicuous were the NADPH-oxidase inhibitor, acetovanillone (apocynin) [53] and the superoxide and hydrogen peroxide scavengers, tiron and catalase, respectively [54,55]. These chemicals almost completely abrogated the *Br*-LPS-induced PMN cell death.

Since inhibition of the check point kinase 1 (Chk1) significantly prevented the cell death of *Br*-LPS treated PMNs, then we explored the induction of DNA damage. One hour after *B*. *abortus* infection, the fragmentation of PMN DNA was already evident (S5A Fig). The DNA damage induced by *B*. *abortus* infection or by *Br*-LPS treatment was reversed by pan-caspase inhibition (S5B Fig), suggesting the participation of caspase-activated DNase (CAD) [56].

Blocking of caspase 5 and to minor extent of caspase 4, prevented cell death; however, specific inhibition of caspases 1 had very little effect. Although related to caspase 1, caspases 5 and 4 have different substrates than caspase 1, and the activation of the former caspases induce cell death independently from the later [57]; therefore not necessarily linked in function. This result, suggests the absence of inflammosome recruitment in the *Brucella* induced PMNs cell death. The modest action of BAPTA/AM and Necrostatin-5 indicates partial involvement of Ca++ and the RIP1 kinase/FADD cell death routes [58].

**Fig 12:**
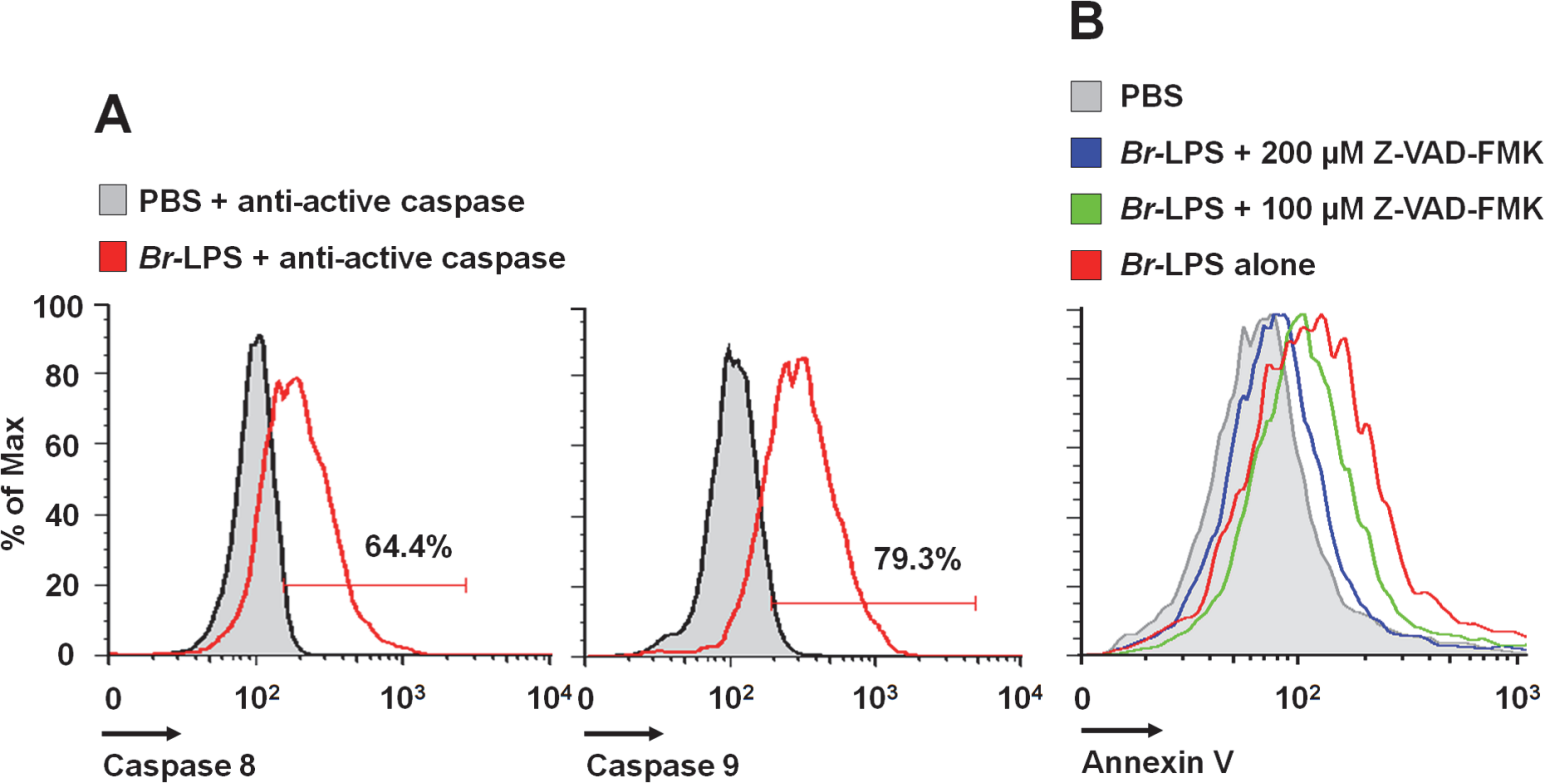
*Br-*LPS induces activation of caspase 8 and 9 in PMNs. (A) Heparinized blood was incubated with 0.3 pmol/mL of *Br-*LPS or PBS for 30 minutes and stained with anti-active caspase 8 or anti-active caspase 9. PMNs population was analyzed by each caspase marker (B) Heparinized blood samples were treated with Z-VAD-FMK or PBS for 1 hour and then incubated with *Br*-LPS (1.5 pmol/mL) for 2 hours. PMNs population was analyzed by Annexin V. Geometric means of histograms are displayed as relative units. Experiments were repeated at least three times.

Caspase-8 and caspase-9 are important mediators of cell death through the extrinsic and intrinsic pathways [59,60]. As shown in Fig 12, both caspases became activated after treatment of PMNs with *Br-*LPS. In spite of this, specific inhibitors for these caspases had little effect in preventing the death of PMNs (Fig 11). This effect was specific for PMNs since caspase triggering was not observed in other blood cells, such as lymphocytes (S3 Fig). This suggests the downstream recruitment of caspases 8 and 9, after the initial cell death triggering mechanisms. Other inhibitors, such as those used for preventing necrosis, apoptosome formation or the activity of Ca^++^ dependent-ATPase or MAP-, tyrosine- or PI3-kinases did not have any effect in blocking the action of *Br*-LPS (Fig 11).

### *Brucella* and *Br-*LPS induce low levels of proinflammatory cytokines in PMNs

**Fig 13:**
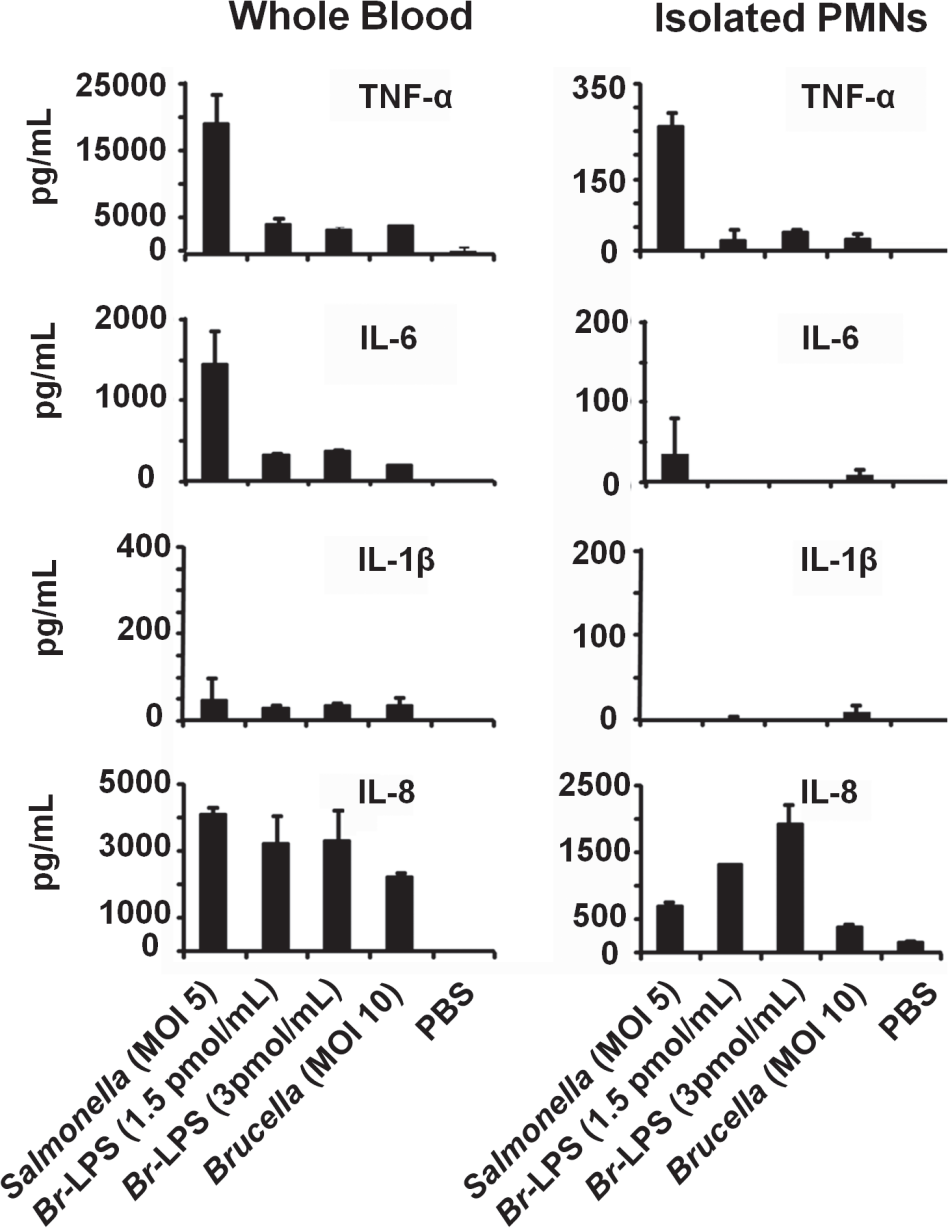
Cytokine differences between blood and purified PMNs infected with *B*. *abortus* or stimulated with *Br*-LPS. The level of the indicated cytokines was determined by ELISA in the plasma of heparinized blood or in the culture supernatants of purified PMNs after treatment with *S*. *enterica*, *B*. *abortus* or *Br-*LPS at various concentrations for two hours. Experiments were repeated at least three times.

Pro-inflammatory TNF-α, IL-1β and IL-6 cytokines and IL-8 chemokine, may influence the life of PMNs, either prolonging or inducing the death of these phagocytic leukocytes [61–64]. Therefore, we assessed the release of these cytokines and chemokines in whole blood cell preparations or purified PMNs after *Brucella* or *Br-*LPS treatment. *S*. *enterica* was included as a control. As shown in Fig 13, there were significant quantitative differences in cytokine production between blood and purified PMNs. *Salmonella* stimulates the release of cytokines by PMNs (Fig 13), induces degranulation and does not prematurely promote the cell death of these cells [65] Regardless whether blood or purified PMNs were tested, the levels of TNF-α, IL-1β and IL-6 were comparatively low after *Brucella* infection or *Br-*LPS treatment. This result is consistent with the low cytokine production by *Brucella* infected or *Br*-LPS treated macrophages [66–69], or by *Brucella* infected mice at early time points of infection [16].

The concentrations of IL-8 induced by *Brucella* or *Br*-LPS in purified PMNs were significantly higher than the levels of the other cytokines (Fig 13). This is striking since it has been established that the chemoattractant IL-8, rather than inducing cell death, promotes PMNs survival [61,70]. Transcription of IL-8 is constitutive in PMNs, making the synthesis of this chemokine readily available after simulation [71]. Still, the cell death remains evident in both PMN preparations, being more conspicuous in blood that in purified PMNs.

## Discussion

The consensus in Gram negative bacterial infections is that the endotoxic LPS molecule and other PAMPs, engage PMNs into activation and prolongation of their life span [63]. This phenomenon is linked to the activation of other cells such as Mϕ, Mo and DCs. In purified PMNs, stimulation of TLR2 and TLR4 with agonists modestly inhibits apoptosis, while in the presence of Mϕ, Mo and DCs, the inhibition of PMN cell death is very potent [47,72]. PMNs are able to use this time delay to recruit other cells and to promote proinflammatory events to eliminate the invading bacteria [73] through actions that involve the respiratory burst [5]. It has been shown that high levels of ROS inhibit caspases activities, suggesting that reactive oxygen species may prevent these proteases from functioning optimally in PMNs [74]. During these processes, some PMNs degranulate, others undergo NETosis, while others may die by necrosis or oncosis, triggering proinflammatory signals [51,52].

In contrast, upon invasion *Brucella* resists the killing action of PMNs and prematurely induces the cell death of these phagocytes. The *Brucella*-induced PMN cell death occurs without bacterial replication [23,24] and without promoting those classical phenotypic changes associated with NETosis, degranulation, necrosis, oncosis or classical apoptosis. The cell death of *Brucella*-infected PMNs seems to be triggered after active phagocytosis of the microorganism followed by the intracellular release of the *Br*-LPS inside cell vacuoles, either by alive or death bacteria. Although the process by which *B*. *abortus* sheds *Br*-LPS inside the cells has not been elucidated, it is likely that it occurs through blebbing of outer membrane fragments enriched in *Br*-LPS, a phenomenon that is well known in *Brucella* [75]. This is significant, since *Br*-LPS is capable to circulate in the body and reside inside phagocytes for months without being destroyed [33], and consequently, capable to exert its biological action on PMNs *in vivo*.

There are reports claiming that *B*. *abortus* and *Brucella* lipoproteins activate PMNs [76]. However, in those experiments the *ex vivo* PMNs viability was less than expected and the assays were performed with heat killed bacteria and lipoproteins acylated in the *E*. *coli* background; thus preventing comparison with our results, as explained before [17]. The interaction between the lipid A of *Br-*LPS and PMNs mostly precludes TLR4, as well as other TLRs, as demonstrated before for Mϕ [18,43,67,77,78]. However, it does not exclude the CD14 molecule, since antibodies against the later co-receptor abrogates the PMN cell death and to less extent the release of TNF-α, suggesting some signaling through this co-receptor. The interaction of *Br*-LPS with intracellular CD14 molecules is feasible, since this lipoprotein is also found inside PMN vesicles [79] and in concordance with the transport of *Br*-LPS to CD14 containing lipid rafts in Mϕ membranes [80]. Moreover, in agreement with our results, it has been shown that in Mϕ, *Brucella* signals through CD14 for the production of low amounts of TNF-α [81]. Finally, the involvement of CD14 in the induction of cell death is not without precedent and it has been demonstrated that direct binding of LPS to CD14 ‒without the concourse of TLR4‒ prompts apoptosis in DCs [48].

We have demonstrated in murine Mϕ that *Br*-LPS follows the classical endocytic pathway used by protein antigens but with a slower kinetics [80]. Then, *Br*-LPS is transported to cellular compartments enriched in MHC-II and recycled to the cell surface, where it forms dense macrodomains. Once in the cell membrane, the *B*r-LPS macrodomains segregate several lipid-raft components and interfere with the MHC-II presentation of peptides to specific CD4+ T cells [80]. The initial release of *Br*-LPS inside PMN phagosomes and its subsequently transit within vacuoles seems to occur by a similar mechanism proposed for Mϕ [82]. Likewise, in some infected PMNs, *Br*-LPS was also observed in cell membrane ruffles-like structures. However, the biogenesis and life span between infected human PMNs and murine Mϕ is rather different: while in the former leukocytes *Brucella* induces premature cell death, in the later it prologues the life span and protects against apoptosis [16,22]. Moreover, the amounts of *Br*-LPS internalized by Mϕ are comparatively much higher than those ingested by PMNs [33]. This difference may be linked to the numbers of CD14 surface molecules present in Mo and Mϕ, which are from 30–40 times more abundant than in PMNs [83]. However, the amounts of intracellular *Br*-LPS available in PMNs at early times of cell infection may be considerably larger than in Mϕ; since the former leukocytes ingest larger number of *Brucella* organisms than the latter, which internalize just a few bacteria [84]. These and other differences make quite difficult to perform a detail experimentation of the intracellular trafficking of *Br*-LPS inside PMNs, and alternative methodological approaches would be required. For the moment, this is beyond of our possibilities.

The dose-dependent *Br-*LPS-induced PMN cell death correlates with a modest but steadily increase of ROS mediated by NADPH oxidase. This seems to be the main triggering mechanisms by which the lipid A of *Br*-LPS induces the premature cell death of human PMNs. It is worth noting that several of the molecular pathways causing PMN cell death are dependent on ROS generation. While large amounts of ROS may inhibit caspases, promote necrosis or cause NETosis [74,85,86], low amounts may induce PMN cell death [52].

DNA damage by oxygen radicals is a well-known phenomenon in a variety of cells, including PMNs [52]; and even small amounts of ROS may induce DNA alterations. The recorded DNA fragmentation of *B*. *abortus* and *Br*-LPS treated PMNs recruited Chk1, a protein that coordinates the DNA damage response at the initiation of cell cycle [87]. Although in other cells inhibition of Chk1 induces apoptosis [87], it is likely that in non-dividing cells ‒such as PMNs‒ this protein has a terminal role and its function is not to arrest the cell cycle, but to promote cell death. The PMN DNA fragmentation was partially reversed by pan-caspase inhibitors; event that suggests the participation of CAD [56].

At first glance, the profile of inhibitory substances suggests that caspase-8 could be extrinsically activated through the RIP1 kinase/FADD route [88]. In addition, the mobilization of Ca^++^ may activate several death signals, including the calcium-activated cysteine protease calpain that cleaves and thereby activates a number of molecules that have important functions in the apoptosis processes [89].

As already recorded in human monocytes [68] the amounts of IL-1β induced by *B*. *abortus* and its *Br*-LPS in PMNs are rather low. In addition, inhibition of caspase 1 did not block the *Br*-LPS mediated PMN cell death. These two observations, together with the low cytokine induction by *Brucella* and its *Br*-LPS in PMNs, seem to preclude the role of the inflammasome pathway in the premature death of these leukocytes. Though this seems relevant, it has been reported that caspase-1 induced pyroptotic cell death does not function in PMNs [90]. Moreover, upon inflammasome activation the amounts of IL-1β produced by purified PMNs are rather low [91]. This may be linked to the fact that human neutrophils express key components of the inflammasome machinery at non-canonical intracellular sites [91]. A general proposal of the mechanisms for the induction of the premature PMN cell death generated during *B*. *abortus* infection is presented in S6 Fig.

It is well known that under certain circumstances, proinflammatory cytokines produced by leukocytes during Gram negative endotoxemia are capable of inducing programmed cell death [92,93]. Among these, the TNF-α is the most conspicuous cytokine generating apoptosis through binding to its cognate TNFR1 [94,95]. However, it is unlikely that TNF-α is the signal that promotes the *Br*-LPS*-*induced PMN cell death. First, the amounts of proinflammatory cytokines ‒including TNF-α‒ produced upon exposure of PMNs to *Brucella* or *Br*-LPS were very low (Fig 13). Second, it is well known that *Brucella* and *Br*-LPS are low agonist of pattern recognition receptors and low activators of NF-κB [16,43,67]. Third, under similar experimental conditions the *Ec*-LPS ‒which induces the production of much higher quantities of TNF-α‒ does not promote premature PMN cell death (Figs 6A and 9). Finally, under the same experimental conditions *Br*-LPS did not induce the death of lymphocytes (Fig 6B) which are also susceptible to the pro-apoptotic effect of TNF-α [95].

It is worth noting that the amounts of IL-8 induced by *B*. *abortus* in purified PMNs were higher than other cytokines. It has been shown that this chemokine, rather that promoting cell death, delays spontaneous and TNF-α-induced apoptosis of human PMNs in a dose dependent manner [70]. The delay in apoptosis is mainly mediated through the interaction of IL-8 with its cognate RII receptor, while the RI receptor may provide an added effect. Still, PMNs died after *Brucella* infection or *Br*-LPS treatment, precluding the influence of IL-8 in a delimited population of PMNs. The different levels of cytokines detected in whole blood versus purified PMNs exposed to *Brucella* or *Br*-LPS may reflect the participation of Mo, DCs and serum components (e.g. complement) present in blood, which may have served as an additional stimuli and sources of cytokines, including IL-8.

For many years it has been recognized that a proportion of patients with chronic brucellosis display absolute neutropenia [27,28]. It has been also shown that the invasion of *Brucella* organisms induces significant hematological chages in the bone marrow, involving pancytopenia and phagocytosis of blood elements (including PMNs) by resident Mϕ [96–98]. In addition, during the accute phase of brucellosis there is a conspicuous absence of infected PMNs in the target organs, a phenomenon that is in clear contrast to the presence of *Brucella* inside Mϕ and DCs [29]. The fact that *Brucella* and its *Br*-LPS specifically induce the premature cell death of PMNs may explain, at least in part, these clinical signs.

Dying PMNs display “eat-me” signals. Therefore, they are readily removed by phagocytic cells. Then, it is likely that *Brucella* infected PMNs may serve as “Trojan horse” vehicles for dispersing the bacterium to other organs; hence, contributing to the long lasting infections observed in brucellosis [99]. The *Brucella*-induced cell death ‒without significant activation of PMNs and their non-phlogistic removal by Mϕ and DCs‒ would help to hamper the promotion of proinflammatory signals (S7 Fig). This mechanism may represent a seminal component of the stealthy strategy used by *Brucella* organisms [16] to spread in its host while avoiding innate immunity.

## Materials and Methods

### Ethics

Human fresh blood was obtained in the blood bank of the Charité Hospital, Berlin, following a protocol approved by the Charité Hospital, Berlin Ethical Committee. Fresh blood was also obtained from normal healthy volunteer donors through the “Etablissement Français du Sang” following their approval and in agreement with the “French Ethics Committee on Human Experimentation F11”, within a convention EFS-08-21-2012 with Institut National de la Santé et de la Recherche Médicale, signed. All blood donors involved were informed about the study and provided written consents.

### Bacterial strains, LPSs and lipid A preparations

Virulent *B*. *abortus* (2308), *B*. *abortus*-GFP (2308) [100], transgenic *B*. *abortus-*RFP (2308) with an integrated chromosomal gene coding for the red fluorescent protein from *Discosoma* coral (provided by Dr. Jean-Jacques Letesson; Unité de Recherche en Biologie Moléculaire, Facultés Universitaires Notre-Dame de la Paix, Namur, Belgium) and *Salmonella enterica* sv. Typhimurium (SL1344) were grown in tryptic Soy or Luria Bertani broths as previously described [16]. Bacterial cells were washed three times by centrifugation in Hanks or PBS solution before the assays. Purified LPSs were prepared from *B*. *abortus* (2308), *B*. *abortus wadC* (2308), *E*. *coli* (0127), *Y*. *enterocolitica* O:9 (MY79), *O*. *anthropi* (LMG 3331T) as reported before [43,77]. *B*. *abortus* lipid A was prepared by mild acid hydrolysis from *Br*-LPS and solubilized as described elsewhere [101]. All the *Brucella Br*-LPS and lipid A preparations were above 98% pure and devoid of contaminant proteins, free lipids and cyclic glucans.

### Neutrophil purification

PMNs were purified by Histopaque and Percoll gradients from blood of healthy donors as previously described [16,50]. Cell preparations were composed from 95–98% of granulocytes. Cell viability was >90%. PMN preparations were maintained at 4°C in PBS or autologous plasma, and used within the first hour after extraction. Under our conditions, PMN spontaneous apoptosis was just evident after 5–7 hours after purification.

### Bactericidal activity

Bactericidal activity was measured as previously described [16]. Briefly, *B*. *abortus* or S. *enterica* were mixed with 500 μL of purified human PMNs (1x10^6^ PMNs/mL) at a MOI of 5 bacteria/PMN and incubated under mild agitation for 90 minutes. Control bacteria were incubated in the absence of PMNs to quantify bacterial replication during the experiment. Viable CFU were determined at 0, 45 and 90 minutes of incubation by lysing cells with 0.1% triton and plating samples in tripticase soy agar. The percentage of bacterial survival was calculated.

### Phagocytosis assay

Human heparinized blood or purified PMNs were incubated with *B*. *abortus*-GFP or fluorescent latex beads for two hours at 37°C a multiplicity of infection (MOI) of 10–100 bacteria or beads/cell, under mild agitation. Smears were fixed with methanol and mounted with ProLong Gold Antifade Reagent with DAPI. One hundred PMNs were counted per sample and the number of particles determined to calculate the percentage of phagocytosis.

### PMN cell death assays

PMN cell death was analyzed by treating human whole blood or isolated PMNs with different bacterial strains and LPSs. Human heparinized blood (100 or 500 μL) collected with lithium heparin was incubated for 2 hours at 37°C in agitation (200–300 rpm) with each treatment. Bacteria were tested at MOIs of 1, 10 or 100 bacteria/PMN. In the case of LPS or lipid A, blood samples were treated at concentrations from 3x10^-3^ to 3x10^1^ pmol/mL. After incubation, blood samples were lysed for 5–10 min in 900 μL of red blood cell lysis buffer (NH_4_Cl 8.02 gm, NaHCO_3_ 0.84gm and EDTA 0.37gm/L, pH 7.2). Cells were washed with ice cold PBS and re-suspended in 100 μL of Annexin V Binding Buffer (BD). 5 μL of Annexin V (BD) and 2 μL of AquaDead (Invitrogen) (diluted 1/20 in PBS) were added and incubated for 30 min on ice in the dark. Cells were washed once with ice cold PBS, re-suspended in 200 μL of paraformaldehyde 3% and incubated for 30 min at room temperature. Samples were then diluted 1:2 with PBS and acquired for analysis within 1 hour.

### Intracellular detection of *Br*-LPS

For intracellular detection of *Br*-LPS a double labeling fluorescence approach was performed [36]. Human heparinized blood was incubated with *B*. *abortus-*RFP (red) for one hour (MOI 2) under mild agitation. Blood smears were fixed and permeabilized with methanol, stained with anti-*Brucella* LPS FITC (green) and mounted with ProLong Gold Antifade Reagent with DAPI (blue). Samples were observed by fluorescent microscopy (Olympus BH-2) under 1000 × magnification. *Br*-LPS shed by *Brucella* is shown in green staining around red bacteria.

Intracellular detection of *Br*-LPS was also performed in *B*. *abortus* infected PMNs by immunogold detection under the electron microscope. Briefly, purified human PMNs 5 ×10^6^ were infected with *B*. *abortus* 2308 at MOI 20. After 1 hour incubation at 37°C under mild agitation, cells were washed and the pellet fixed with 200 μl of 2.5% glutaraldehyde in phosphate buffer 0.05M pH 7.4 (PB) at 4°C for 1 hour. Cells were pelleted at 3000 rpm for 10 min, washed in PB and suspended in 50 μL of PB. Then fixed cells were incubated at 40°C for 5 minutes, and 100 μL of 3% low melting agarose at 40°C added. The temperature was lowered, and 5 volumes of 2.5% glutaraldehyde in PB were added to the solid agarose block and incubated overnight at 4°C. Agarose blocks containing the fixed infected PMNs were processed for inclusion in Spurr resin for immunogold staining and for electron microscopy as described elsewhere [102]. For detection of *Br*-LPS, human IgG or mouse IgG with specificity against the O chain polysaccharide [103] were used in combination with protein-A/protein-G colloidal gold 15 nm (EY Laboratories, Inc.). Purified mouse and human IgGs from normal serum were used for controlling the specificity of the reaction. Finally, PMNs sections were stained following the led citrate procedure described by Reynolds [104] [101] and observed under a Hitachi H 7100 electron microscope.

### Quantitation of *Br*-LPS interacting with PMNs

In order to determine the amount of *Br*-LPS interacting with human PMNs, 10 μg (0.3 pmol) of *Br*-LPS were incubated with 1x10^6^ PMNs in 500 μL of HBSS at 37°C for 1 h under mild rotation in the presence or absence of human IgG anti-*Br*-LPS. PMNs were washed three times with HBSS to remove the excess of *Br*-LPS, and then the cell pellet lysed with deionized water containing 50 μg/mL of DNAase and 125 μg/mL of proteinase K (Fisher Scientific) at 37°C for 1 h under mild rotation. Cell lysate was incubated with SDS-PAGE sample buffer and subjected to Western blotting. *Br*-LPSs bands were revealed with a monoclonal antibody against the O chain polysaccharide of the *Br*-LPS conjugated with peroxidase [105]. Controls included the assay performed with *Br*-LPS in the absence or presence of human antibodies but in the absence of PMNs, and PMNs alone. Quantitation of *Br*-LPS bound to PMNs was estimated in relation to a standard curve of purified *Br*-LPS ranging from 0.1 ng to 12 ng. The read-out of the bands was performed by densitometry with the support of ImageJ software (http://imagej.net/).

### ROS detection

Isolated PMNs (1x10^5^) were re-suspended in 50 μL of Hanks Balanced Salt Solution (HBSS+1% FBS) per well of a 96-uncoated serum well plate. Cell suspension was supplemented with Reactive Oxygen Species (ROS) Detection Reagents (Invitrogen) and stimulated with phorbol myristate acetate (40 nM), *Br-*LPS (0.03–3 pmol/mL), *Ec-*LPS (0.09–7.5 pmol/mL) in 50 μl HBSS+1% FBS or left untreated. The kinetics of ROS production was monitored with a Victor Perkin Elmer luminometer at 37C for 90 min.

### NET formation and cell cytotoxicity assay

Isolated PMNs (1x10^5^) were re-suspended in 500 μL of RPMI medium (10 mM HEPES +1% FBS without glutamine) and let sit on 24-well plates for 30 min at 37°C. Cells were stimulated with phorbol myristate acetate (40 nM) (Sigma), *Br-*LPS (0.7–100 μg/ml), *Ec-*LPS (0.7–100 μg/ml) or left untreated in RPMI medium. After 6h 30 minutes, cell cytotoxicity was measured by Sytox (0.3 μM) (Invitrogen) staining with a fluorometer. Some cells were fixed in paraformaldehyde 8% and observed with a Leica inverted fluorescence microscope to evaluate the nuclear morphologies and NET spreading.

### Cytokine quantitation

The levels of TNF-α, IL-8, IL-1β and IL-6 were measured by ELISA (eBioscience) in heparinized human blood (plasma) or supernatant of isolated PMNs treated with different stimuli according to manufacturer’s specifications.

### Determination of caspase 8 and 9 activation

Heparinized human blood (500 μL) was incubated with *B*. *abortus* LPS (10 μg/mL) or PBS for 30 minutes under mild agitation and stained directly and incubated with anti-active caspase 8 or anti-active caspase 9 using Guava Caspase 8 FAM & Caspase 9 SR Kit (Millipore) according to manufacturer’s specifications and quantitated by flow cytometry. PMNs population was gated by forward light scatter and side light scatter parameters and analyzed by each caspase marker.

### DNA fragmentation assays

PMNs were isolated as previously described [106] and incubated for one hour with *B*. *abortus* (MOI 100) or 10 μg/mL (0.3 pmol) of *Br-*LPS in the presence or absence of a pan-caspase inhibitor (Z-VAD-FMK). Cycloheximide (Sigma-Aldrich) was used as a positive control for DNA fragmentation. After incubation, PMN cell death was measured by using a DNA fragmentation ELISA (Roche) according to manufacturer’s specifications. For microscopic analysis, heparinized blood was incubated with *B*. *abortus-*RFP for 2 hours (MOI 100). Red blood cells were lysed and total leucocytes prepared, fixed and stained with APO-BrdU TUNEL Assay Kit (Invitrogen) according to manufacturer’s specifications. Cells were centrifuged on a microscope slide by using a Cytospin 2 (Shandon) and mounted with ProLong Gold Antifade Reagent with DAPI (Invitrogen).

### TLR4 and CD14 neutralization

TLR4 and CD14 cell receptors were neutralized (before *Br-*LPS or *Ec-*LPS treatments) by incubating isolated PMNs or heparinized human blood with 1 μg of anti-hTLR4-IgG (clone W7C11) or 5 μg of anti-CD14-IgA (clone D3B8) antibodies (InvivoGen), for 20 minutes and one hour respectively. No inhibitory or stimulatory signals were observed with mouse monoclonal IgG1 (anti-bovine IgG, Sigma-Aldrich), or enriched mouse and human immunoglobulin preparations. Receptor blockage was verified in side controls by measuring TNF-α secretion after treating blood with 5 μg/mL *Ec-*LPS (Fig 9A).

### PMN cell death inhibition assays

Heparinized human blood samples (350 μL) were pre-incubated with one of the following compounds for 1 hour: IM-54 (Enzo Life Sciences), Wortmanin, Genistein, Tyrphostin, PD098059, Necrostatin-5, Z-VAD-FMK, AZD7762, Catalase, Tiron, Acetovanillone (Sigma), BAPTA/AM, YVAD-CHO, NS3694, Thapsigargin (Calbiochem), Z-IETD-FMK, Z-WEHD-FMK, Z-LEVD-FMK, Z-LEHD-FMK (BioVision), Z-YVAD-FMK (Santa Cruz). Concentrations were utilized according to previous reports and standardized to our conditions for optimal inhibitory performance. After treatment with the inhibitory compounds, samples were incubated with *Br*-LPS (1.3 pmol/mL) for 2 hours. Samples were further processed and analyzed by cytometry for cell death with Annexin V as described above.

### Flow cytometry and FACS analysis

PMN or lymphocyte populations were gated as indicated (S8 Fig) and analyzed for cell death of caspase activation by flow cytometry. FACS analysis was performed using a FACSCanto system (BD Biosciences) or Guava easyCyte (Millipore). FACS data were analyzed using FlowJo software (Tree Star, Inc.). For each experiment, control samples were included to define the proper gates.

### Statistical analysis

Values were expressed as means ± standard error, and compared using Student’s t test for determining the statistical significance in the different assays. Values of *p*< 0.05 were considered statistically significant.

## Supporting Information

S1 Fig Schematic structure of smooth *B*. *abortus Br-*LPS. The O-polysaccharide is an unbranched linear homopolymer of α-1,2-linked 4,6-dideoxy-4-formamido-D-mannopyranosyl units (*N*-formylperosamine) with an average chain length of 96 to 100 glycosyl subunits [105]. The O-polysaccharide is linked to a core bifurcating oligosaccharide composed of βGlcN-6-βGlcN-4-βGlcN(-6-βGlcN)-3-αMan(-6-αGlc)-5-KDO1(-2-KDO2)-Lipid A; branching from KDO1 is αPerNFo-[-2PerNFo]_n_-2PerNF-2-αMan-3-αMan-3-βQuiNAc-4-βGlc-4-KDO2-4-KDO1 [44]. The KDO1 is linked to the lipid A composed of a backbone of diaminoglucose (DAG) disaccharide, substituted with phosphates (P) and amide and ester-linked long chain saturated (C_16:0_ to C_18:0_) and hydroxylated (3-OH-C_12:0_ to 29-OH-C_30:0_) fatty acids [42,107]. Ketodeoxyoctulosonic acid (KDO), mannose (Man), Acetyl-quinovosamine (QuiN), glucose (Glc).
(TIF)

S2 Fig Neutralization of TLR4 does not protect against *Br-*LPS-induced PMN cell death. Heparinized blood was incubated with *Br-*LPS (3 pmol/mL) alone or previously neutralized with anti-TLR4 and PMN population gated and analyzed by Annexin V marker. Geometric means of histograms displayed as relative units. Experiments were repeated at least three times.
(TIF)

S3 Fig *Br-*LPS induces little activation of caspase 8 and 9 in lymphocytes. Heparinized blood was incubated with 0.3 pmol/mL of *Br-*LPS or PBS for 30 minutes and stained with anti-active caspase 8 or anti-active caspase 9. Lymphocyte population was gated by forward light scatter and side light scatter parameters and analyzed for each caspase marker. Geometric means of histograms are displayed as relative units. Experiments were repeated at least three times.
(TIF)

S4 Fig Cell death promoted by *Br-*LPS failed to induce NETosis. Isolated PMNs were stimulated with PMA (40nM) or *Br*-LPS (3 pmol/mL). (A) NET formation induced by PMA, or (B) cell cytotoxicity induced by *Br*-LPS was analyzed under the fluorescent microscope. (C) Cell morphology of PMA treated cells, or (D) *Br*-LPS treated cells were observed using phase contrast. NET formation is clearly seen in “A”, while in “B” cell death without NET formation is observed. Microscope images are at 400 × magnification. Figure represents the outcome of a single experiment. Similar results were obtained in repeated experiments by looking NET spreading
(TIF)

S5 Fig *Brucella* and *Br-*LPS induces PMNs DNA fragmentation. (A) Heparinized blood was incubated with *B*. *abortus-*RFP for 2 hours (MOI 100). Red blood cells were lysed and total leucocytes prepared, fixed and stained with APO-BrdU TUNEL Assay Kit according to manufacturer’s specifications. Cells were centrifuged and mounted with ProLong Gold Antifade Reagent with DAPI. (a) *B*. *abortus-*RFP, (b) PMN DAPI staining (c) TUNEL positive nucleus and (d) merged images. Images were cut from microscope field, contrasted and saturated using Hue tool to obtain suitable color separation. Images were then merged using Adobe Photoshop 8 software. Microscope images are at 1000 × magnification. (B) Purified blood PMNs were incubated with *B*. *abortus* (MOI 100) or *Br-*LPS (0.3 pmol/mL) in the presence or absence of a pan-caspase inhibitor (Z-VAD-FMK) for one hour. Cycloheximide was used as a positive control. PMN DNA fragmentation was measured by Cellular DNA Fragmentation ELISA (Roche). Values of *p*<0.01 (**) are indicated.
(TIF)

S6 Fig Proposed model for the premature cell death of Brucella infected PMNs. After *Brucella* invasion, the bacterium is readily phagocytized by resident PMNs [24] resisting the killing mechanisms mediated by these leukocytes [31]. Once inside phagosomes, the bacterium releases non-toxic *Br*-LPS, probably in the form of outer membrane fragments [75]. Then, the *Br*-LPS fuses with the cell membrane of PMNs, binds to CD14 lipoprotein and is transported inside the cytoplasm of PMNs within endocytic vacuoles. During this process, the *Br*-LPS does not interact with TLR-4; then, avoiding activation of PMNs. In the course of this action, NADPH oxidase is progressively recruited promoting the slow generation of controlled amounts of ROS mediators. These effectors induce oxidative damage of nuclear DNA inducing molecular fragmentation and the recruitment of Chek1 protein, which is the main responsible for coordinating the DNA damage response at the initiation of the cell cycle. In PMNs ‒which are non-dividing effector cells‒ Chek1, rather than arresting the cell cycle, may recruit cell death executioner caspases which in course promote the activation of caspase-activated Dnases (CAD), contributing to the damage of DNA. At the same time, some of the ROS effectors may act as second messengers and induce the activation of caspases 5 and to minor extend caspase 4, but not caspase 1, excluding the participation of the inflammasome pathway. ROS may also induce the recruitment of the RIP1 kinase/FADD cell death routes, caspase 8 and promote the release of Ca^++^ to the cytosol. These mediators, will also recruit cell death executioner caspases and together with ROS mediators trigger additional death effector mechanisms (e.g. activation of calpains and cathepsins). Finally, the activation of the initiator caspase 9 of the intrinsic cell death pathway will be activated downstream by caspase 8 contributing to the premature PMN cell death mechanism. During this process, the infected PMNs expose “eat-me” signals (e.g. phosphatidylserine) on the surface that promote their phagocytosis by Mϕ or DCs.
(TIF)

S7 Fig PMN cell death modulation. After danger signal or PAMP recognition, PMNs become activated, cell death delayed and inflammatory response promoted. Under noninfectious conditions, PMNs die spontaneously and are phagocytized by DCs and MØ under non-inflammatory conditions. Following PMNs ingestion of *Brucella*, PMNs are quickly primed for cell death and phagocytized by DCs and MØ where *Brucella* replicates intensively under a non-inflammatory environment.
(TIF)

S8 Fig PMNs and lymphocytes gating strategy. (A) PMN or lymphocyte cell populations were gated by forward light scatter and side light scatter parameters from total blood leucocyte population. (B) GFP negative or GFP positive population (infected with *B*. *abortus-*GFP) were selected and (C) analyzed for cell death by AquaDead and Annexin V markers.
(TIF)
