# Supplementary figures and images for: Brucella abortus Induces the Premature Death of Human Neutrophils through the Action of Its Lipopolysaccharide
Source: PLoS Pathog. 2015 May 6;11(5):e1004853. doi: 10.1371/journal.ppat.1004853 (PMC4422582; doi:10.1371/journal.ppat.1004853)

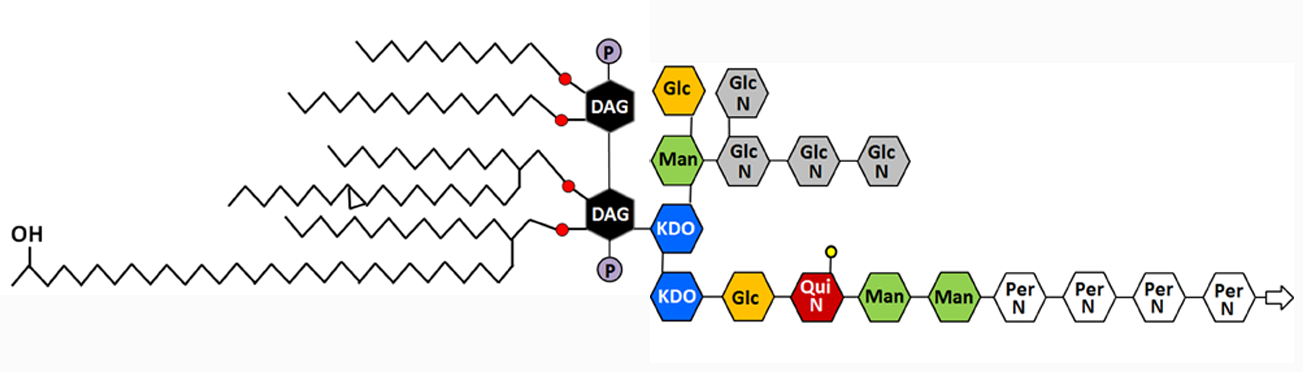

Supplement: S1 Fig — The O-polysaccharide is an unbranched linear homopolymer of α-1,2-linked 4,6-dideoxy-4-formamido-D-mannopyranosyl units (N-formylperosamine) with an average chain length of 96 to 100 glycosyl subunits [105]. The O-polysaccharide is linked to a core bifurcating oligosaccharide composed of βGlcN-6-βGlcN-4-βGlcN(-6-βGlcN)-3-αMan(-6-αGlc)-5-KDO1(-2-KDO2)-Lipid A; branching from KDO1 is αPerNFo-[-2PerNFo]n-2PerNF-2-αMan-3-αMan-3-βQuiNAc-4-βGlc-4-KDO2-4-KDO1 [44]. The KDO1 is linked to the lipid A composed of a backbone of diaminoglucose (DAG) disaccharide, substituted with phosphates (P) and amide and ester-linked long chain saturated (C16:0 to C18:0) and hydroxylated (3-OH-C12:0 to 29-OH-C30:0) fatty acids [42,107]. Ketodeoxyoctulosonic acid (KDO), mannose (Man), Acetyl-quinovosamine (QuiN), glucose (Glc). (TIF) [file ppat.1004853.s001.tif]

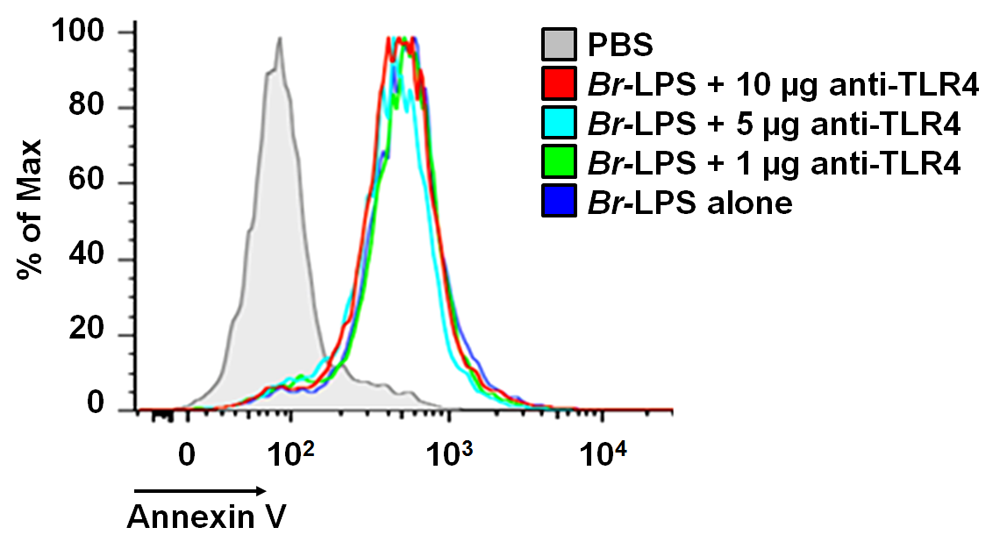

Supplement: S2 Fig — Heparinized blood was incubated with Br-LPS (3 pmol/mL) alone or previously neutralized with anti-TLR4 and PMN population gated and analyzed by Annexin V marker. Geometric means of histograms displayed as relative units. Experiments were repeated at least three times. (TIF) [file ppat.1004853.s002.tif]

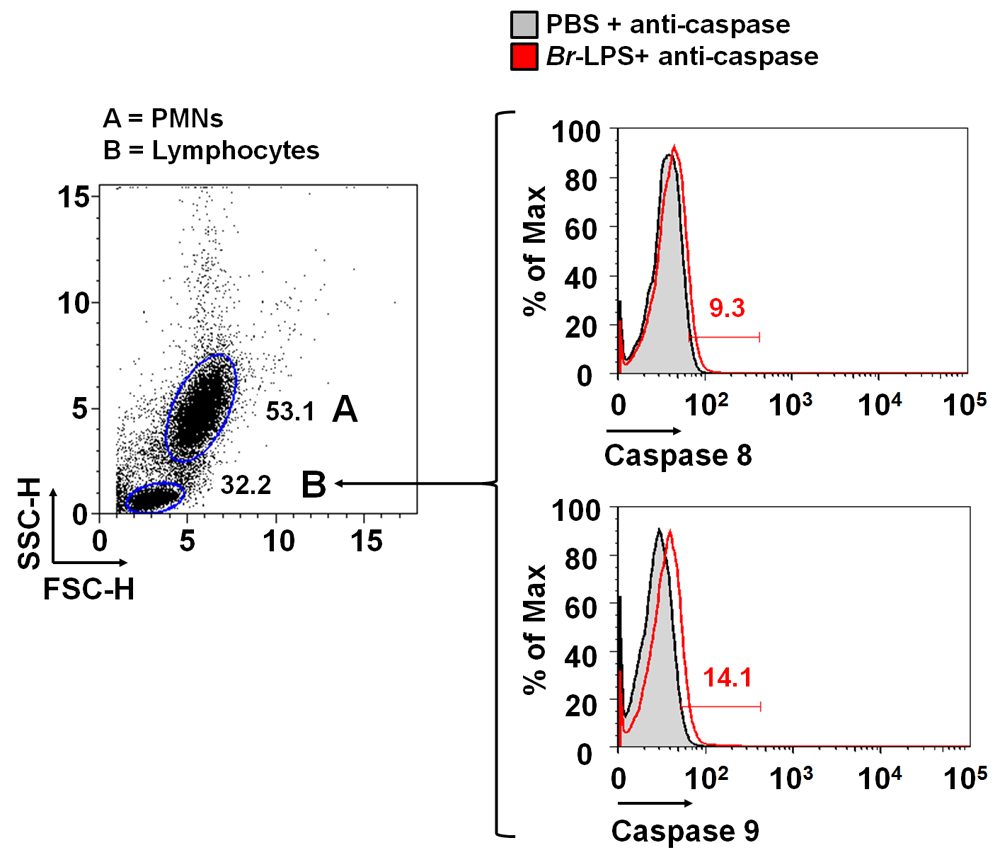

Supplement: S3 Fig — Heparinized blood was incubated with 0.3 pmol/mL of Br-LPS or PBS for 30 minutes and stained with anti-active caspase 8 or anti-active caspase 9. Lymphocyte population was gated by forward light scatter and side light scatter parameters and analyzed for each caspase marker. Geometric means of histograms are displayed as relative units. Experiments were repeated at least three times. (TIF) [file ppat.1004853.s003.tif]

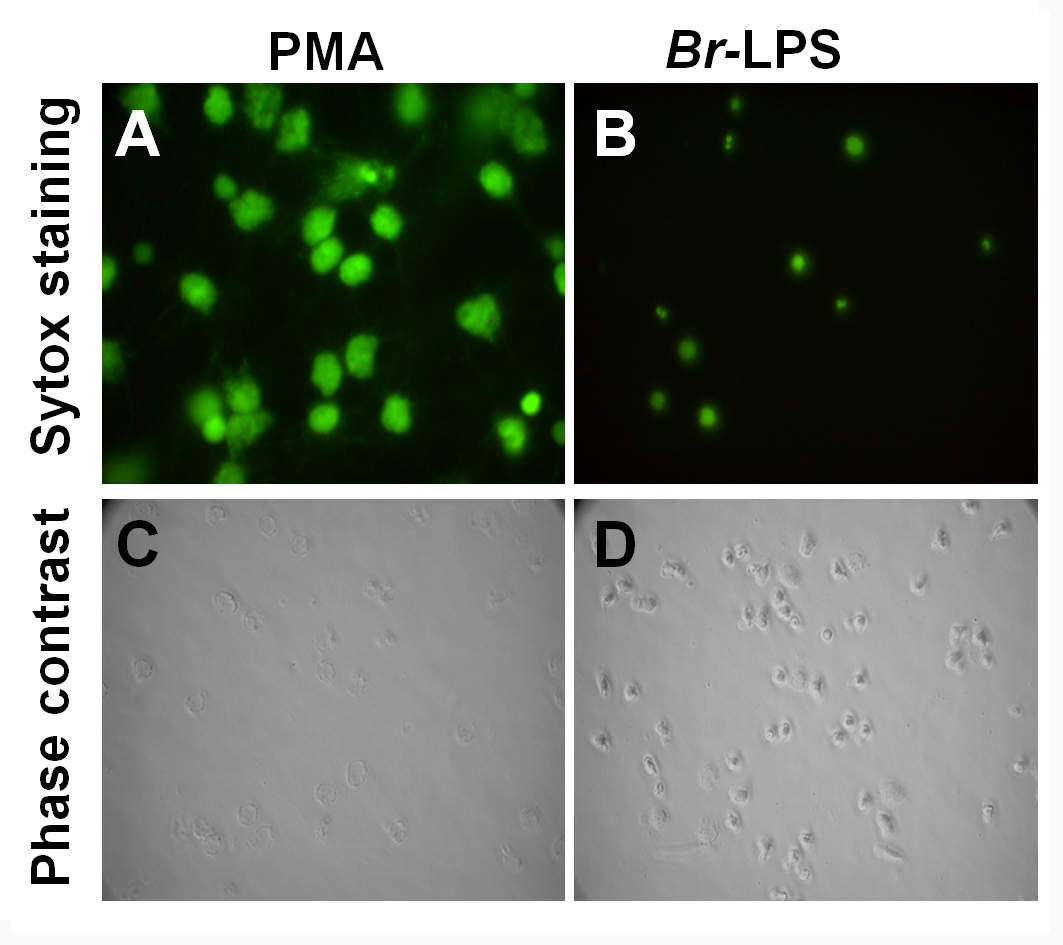

Supplement: S4 Fig — Isolated PMNs were stimulated with PMA (40nM) or Br-LPS (3 pmol/mL). (A) NET formation induced by PMA, or (B) cell cytotoxicity induced by Br-LPS was analyzed under the fluorescent microscope. (C) Cell morphology of PMA treated cells, or (D) Br-LPS treated cells were observed using phase contrast. NET formation is clearly seen in “A”, while in “B” cell death without NET formation is observed. Microscope images are at 400 × magnification. Figure represents the outcome of a single experiment. Similar results were obtained in repeated experiments by looking NET spreading (TIF) [file ppat.1004853.s004.tif]

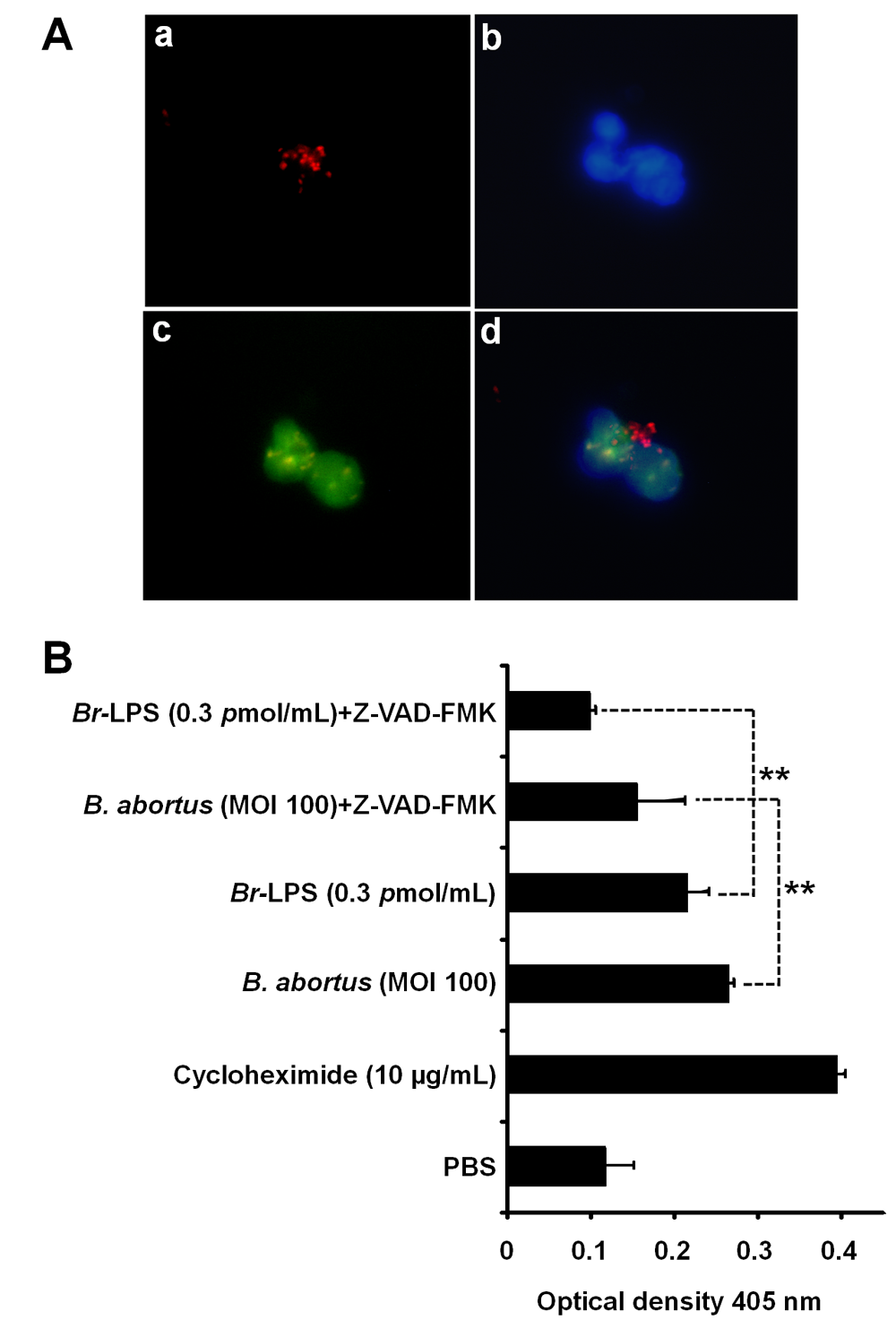

Supplement: S5 Fig — (A) Heparinized blood was incubated with B. abortus-RFP for 2 hours (MOI 100). Red blood cells were lysed and total leucocytes prepared, fixed and stained with APO-BrdU TUNEL Assay Kit according to manufacturer’s specifications. Cells were centrifuged and mounted with ProLong Gold Antifade Reagent with DAPI. (a) B. abortus-RFP, (b) PMN DAPI staining (c) TUNEL positive nucleus and (d) merged images. Images were cut from microscope field, contrasted and saturated using Hue tool to obtain suitable color separation. Images were then merged using Adobe Photoshop 8 software. Microscope images are at 1000 × magnification. (B) Purified blood PMNs were incubated with B. abortus (MOI 100) or Br-LPS (0.3 pmol/mL) in the presence or absence of a pan-caspase inhibitor (Z-VAD-FMK) for one hour. Cycloheximide was used as a positive control. PMN DNA fragmentation was measured by Cellular DNA Fragmentation ELISA (Roche). Values of p<0.01 (**) are indicated. (TIF) [file ppat.1004853.s005.tif]

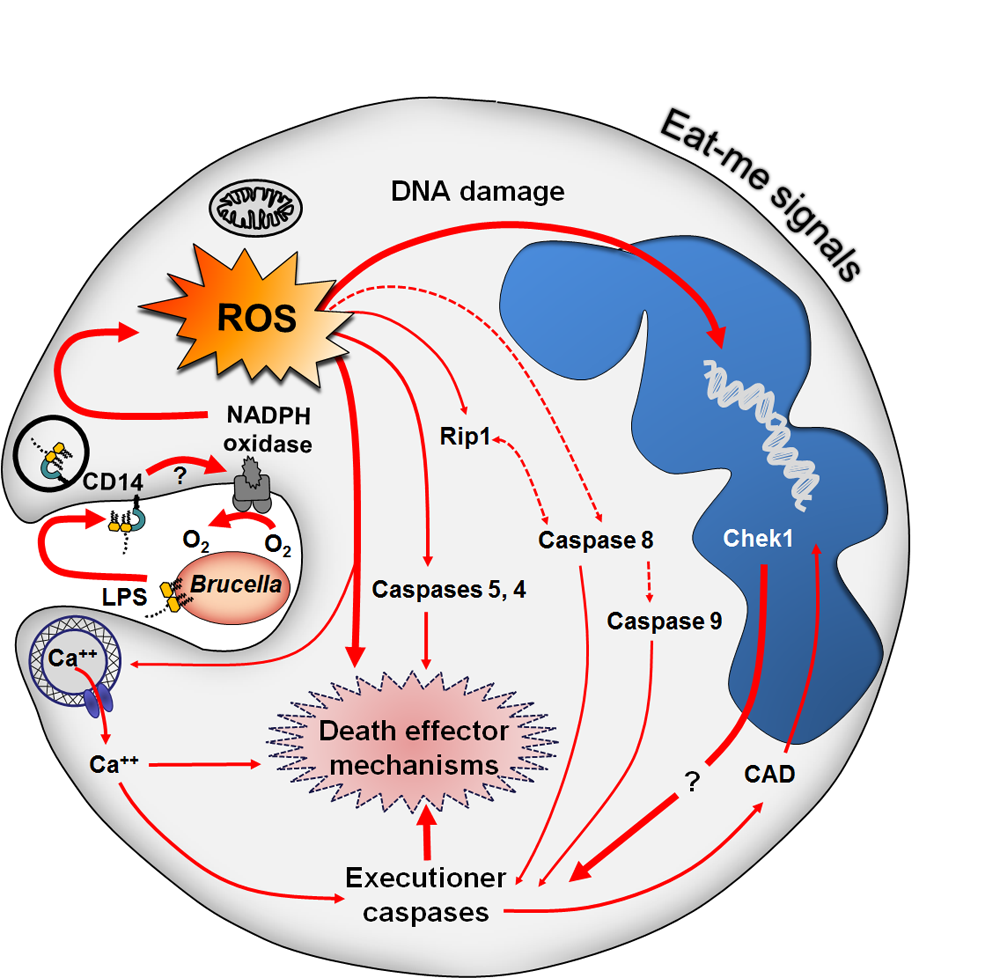

Supplement: S6 Fig — After Brucella invasion, the bacterium is readily phagocytized by resident PMNs [24] resisting the killing mechanisms mediated by these leukocytes [31]. Once inside phagosomes, the bacterium releases non-toxic Br-LPS, probably in the form of outer membrane fragments [75]. Then, the Br-LPS fuses with the cell membrane of PMNs, binds to CD14 lipoprotein and is transported inside the cytoplasm of PMNs within endocytic vacuoles. During this process, the Br-LPS does not interact with TLR-4; then, avoiding activation of PMNs. In the course of this action, NADPH oxidase is progressively recruited promoting the slow generation of controlled amounts of ROS mediators. These effectors induce oxidative damage of nuclear DNA inducing molecular fragmentation and the recruitment of Chek1 protein, which is the main responsible for coordinating the DNA damage response at the initiation of the cell cycle. In PMNs ‒which are non-dividing effector cells‒ Chek1, rather than arresting the cell cycle, may recruit cell death executioner caspases which in course promote the activation of caspase-activated Dnases (CAD), contributing to the damage of DNA. At the same time, some of the ROS effectors may act as second messengers and induce the activation of caspases 5 and to minor extend caspase 4, but not caspase 1, excluding the participation of the inflammasome pathway. ROS may also induce the recruitment of the RIP1 kinase/FADD cell death routes, caspase 8 and promote the release of Ca++ to the cytosol. These mediators, will also recruit cell death executioner caspases and together with ROS mediators trigger additional death effector mechanisms (e.g. activation of calpains and cathepsins). Finally, the activation of the initiator caspase 9 of the intrinsic cell death pathway will be activated downstream by caspase 8 contributing to the premature PMN cell death mechanism. During this process, the infected PMNs expose “eat-me” signals (e.g. phosphatidylserine) on the surface that promote thei [file ppat.1004853.s006.tif]

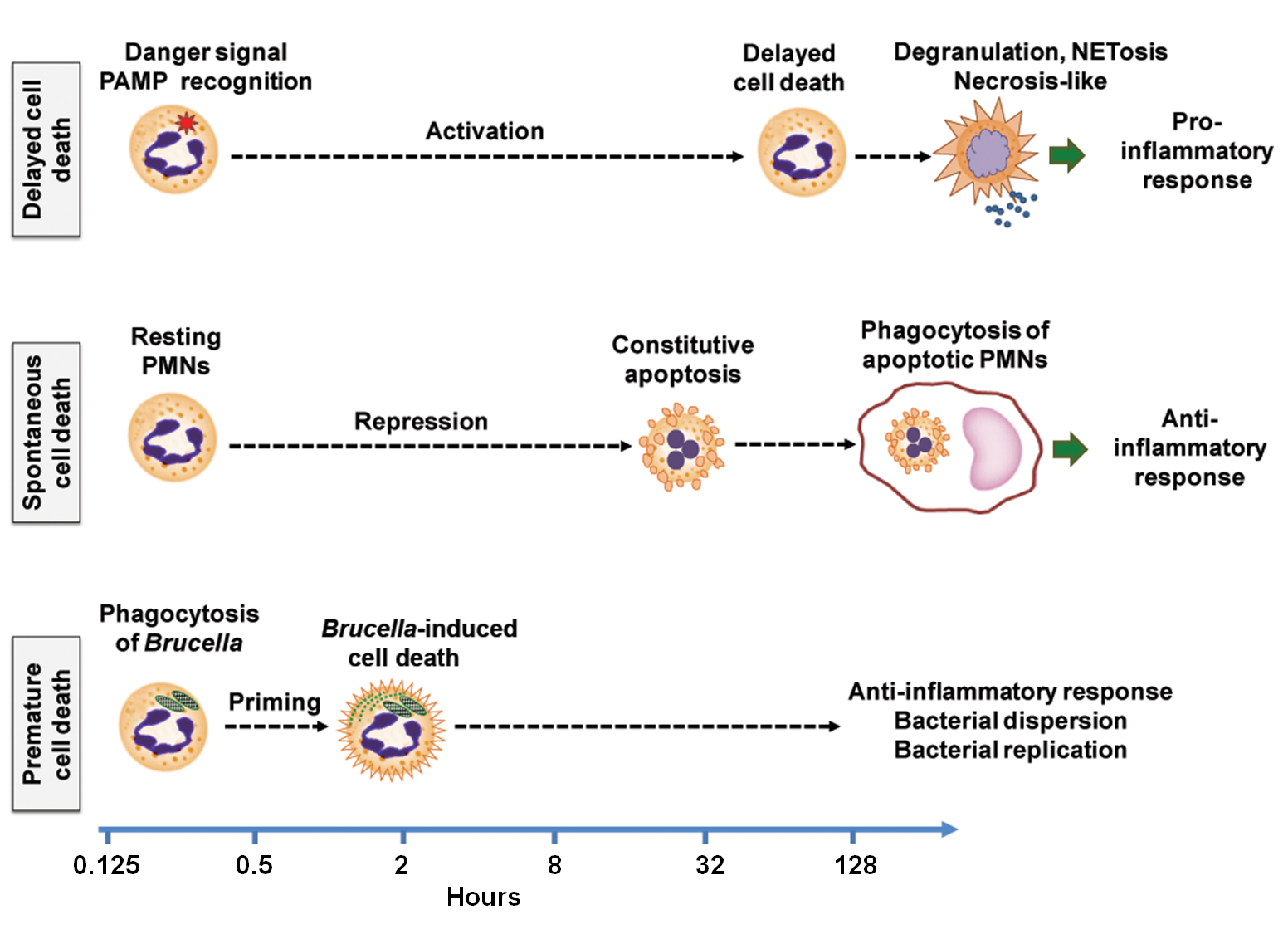

Supplement: S7 Fig — After danger signal or PAMP recognition, PMNs become activated, cell death delayed and inflammatory response promoted. Under noninfectious conditions, PMNs die spontaneously and are phagocytized by DCs and MØ under non-inflammatory conditions. Following PMNs ingestion of Brucella, PMNs are quickly primed for cell death and phagocytized by DCs and MØ where Brucella replicates intensively under a non-inflammatory environment. (TIF) [file ppat.1004853.s007.tif]

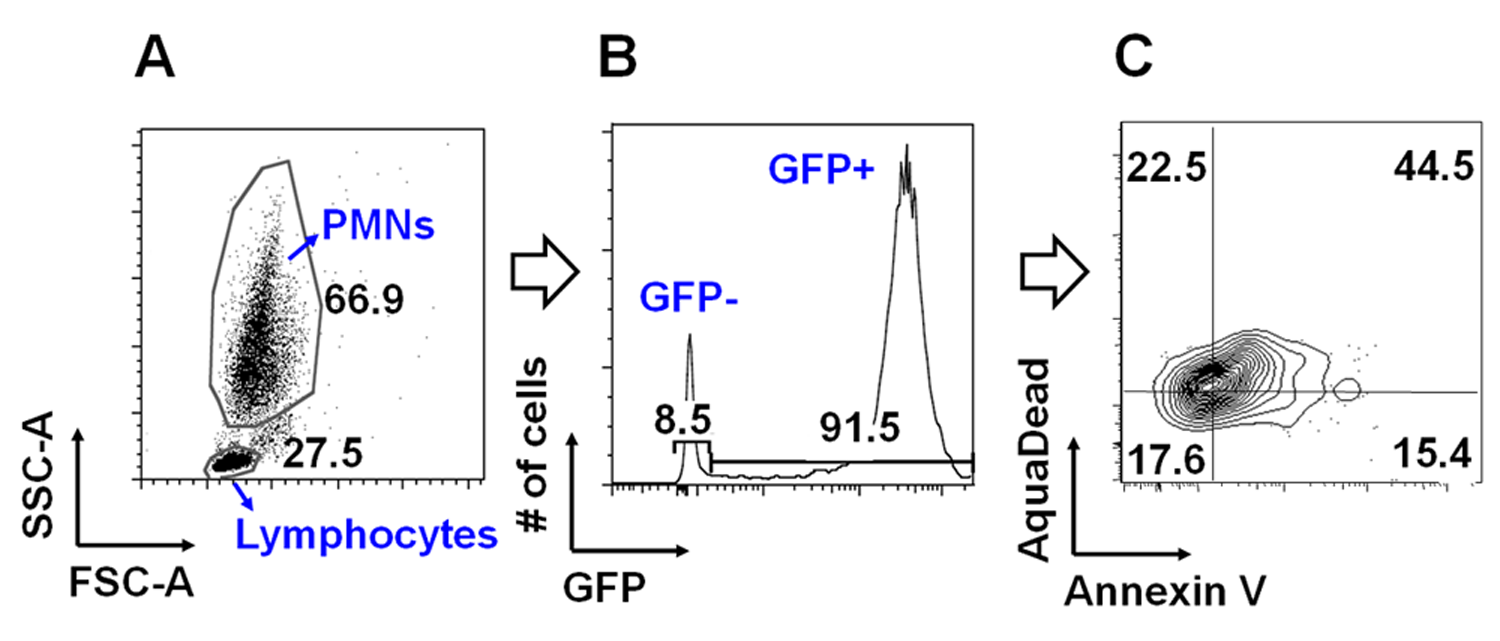

Supplement: S8 Fig — (A) PMN or lymphocyte cell populations were gated by forward light scatter and side light scatter parameters from total blood leucocyte population. (B) GFP negative or GFP positive population (infected with B. abortus-GFP) were selected and (C) analyzed for cell death by AquaDead and Annexin V markers. (TIF) [file ppat.1004853.s008.tif]
